# SMYD3 associates with the NuRD (MTA1/2) complex to regulate transcription and promote proliferation and invasiveness in hepatocellular carcinoma cells

**DOI:** 10.1186/s12915-022-01499-6

**Published:** 2022-12-27

**Authors:** Yang Yang, Rongfang Qiu, Siyu Zhao, Lin Shen, Bufu Tang, Qiaoyou Weng, Ziwei Xu, Liyun Zheng, Weiqian Chen, Gaofeng Shu, Yajie Wang, Zhongwei Zhao, Minjiang Chen, Jiansong Ji

**Affiliations:** 1 Key Laboratory of Imaging Diagnosis and Minimally Invasive Intervention Research, Imaging Diagnosis and Interventional Minimally Invasive Institute, The Fifth Affiliated Hospital of Wenzhou Medical University, Lishui, 323000 China; 2 Department of Interventional Radiology, Clinical College of The Affiliated Central Hospital, Lishui University, Lishui, 323000 China

**Keywords:** SMYD3, Hepatocellular carcinoma, NuRD complex, IGFBP4, Metastasis

## Abstract

**Background:**

SMYD3, a member of the SET and MYND domain-containing (SMYD) family, is a histone methyltransferase (HMT) and transcription factor that plays an important role in transcriptional regulation in human carcinogenesis.

**Results:**

Using affinity purification and mass spectrometry assays to identify SMYD3-associated proteins in hepatocellular carcinoma (HCC) cells, we found several previously undiscovered SMYD3-interacting proteins, including the NuRD (MTA1/2) complex, the METTL family, and the CRL4B complex. Transcriptomic analysis of the consequences of knocking down SMYD3, MTA1, or MTA2 in HCC cells showed that SMYD3/NuRD complex targets a cohort of genes, some of which are critically involved in cell growth and migration. qChIP analyses showed that SMYD3 knockdown led to a significant reduction in the binding of MTA1 or MTA2 to the promoters of IGFBP4 and led to a significant decrease in H4K20me3 and a marked increase in H4Ac at the IGFBP4 promoter. In addition, we demonstrated that SMYD3 promotes cell proliferation, invasion, and tumorigenesis in vivo and in vitro and found that its expression is markedly upregulated in human liver cancer. Knockdown of MTA1 or MTA2 had the same effect as knockdown of SMYD3 on proliferation and invasion of hepatocellular carcinoma cells. Catalytic mutant SMYD3 could not rescue the phenotypic effects caused by knockdown of SMYD3. Inhibitors of SMYD3 effectively inhibited the proliferation and invasiveness of HCC cells.

**Conclusions:**

These findings revealed that SMYD3 could transcriptionally repress a cohort of target genes expression by associating with the NuRD (MTA1/2) complex, thereby promoting the proliferation and invasiveness of HCC cells. Our results support the case for pursuing SMYD3 as a practical prognostic marker or therapeutic target against HCC.

**Supplementary Information:**

The online version contains supplementary material available at 10.1186/s12915-022-01499-6.

## Background

Liver cancer is the seventh most common cancer and the second most common cause of cancer-related death worldwide, killing approximately 830,000 people each year [1]. The prognosis for liver cancer is poor. After pancreatic cancer, liver cancer is the second deadly tumor, with a 5-year survival of just only 18% [2]. The outlook is even grimmer for patients in China, where 5-year survival rates are reported to be as low as 12% [2]. Most patients are diagnosed at an intermediate-advanced stage underlying liver dysfunction, missing the best opportunity for hepatectomy [3]. Only 5 to 15% of patients are eligible for surgical removal, and this applies only to patients in the early-stage, because the hepatic regenerative capacity is reduced, and usually only to those without cirrhosis. Hepatocellular carcinoma (HCC) accounts for approximately 90% of all primary liver cancers. The development of hepatocellular carcinoma is a complex biological process with multiple steps, in which genomic changes induce the formation of cellular intermediates through gradual changes in hepatocellular phenotype, leading to the evolution of HCC [4]. Therefore, there is an urgent need to understand the mechanism of HCC at the molecular level and identify novel potential biomarkers that will significantly improve early diagnosis and provide a new molecular target for clinical treatment.

The SET and MYND domain-containing (SMYD) family of lysine methyltransferases, which include SMYD1–5 five family members, is abundant in both the cytoplasm and nucleus. It is characterized by a cleaved SET methyltransferase catalytic domain, including a zinc finger myeloid-nervy-DEAF1 (MYND) domain, and harbors at least one C-terminal tetratricopeptide repeat (TPR) domain. Both the MYND and TPR domains are responsible for protein–protein interactions [5]. SMYD3 is a member of the family that catalyzes both dimethylation and trimethylation of histone H3 at lysine 4 (H3K4) [6]. H3K4me2 and H3K4me3 near the promoter regions of target genes usually serve as transcriptional activation markers [7, 8]. Many oncogenes have been demonstrated to be regulated by SMYD3 at the transcriptional level through H3K4me3 modification, highlighting that SMYD3 as an important epigenetic regulator in cancer cells [7]. In addition to its H3K4 methyltransferase activity, SMYD3 can also catalyze the methylation of histones H4K5, H4K20, and H2A.Z, with H4K5 and H4K20 being repressive markers [9, 10]. Besides, SMYD3 can methylate nonhistone proteins as well, such as VEGFR1, MAP3K2, AKT1, ER, and HER2 [11–14]. SMYD3 also plays a critical role in cardiac and skeletal muscle development [15]. SMYD3 knockdown resulted in cardiac defects and pericardial edema in zebrafish embryos, indicating that SMYD3 plays a critical role in cardiac development [16]. SMYD3 overexpression significantly increased the expression of genes associated with myogenesis, such as *Mck*, *Mymk*, and *Tnnc1*, and promoted muscle differentiation and myotube fusion in C2C12 murine myoblasts [17]. In addition, SMYD3 plays an important role in tumorigenesis, including in colorectal cancer, hepatocellular carcinoma, breast cancer, gastric carcinoma, cervical cancer, prostate cancer, pancreatic cancer, lung cancer, ovarian cancer, esophageal cancer, bladder cancer, and malignant glioma [14]. The oncogenic phenotypes of SMYD3 include promoting epithelial-mesenchymal transition (EMT), cell cycle alteration, cell proliferation, invasion, metastasis, increased telomerase activity, and cell immortalization [18–22]. Recent studies have indicated that SMYD3 is associated with liver carcinogenesis in mice and poor prognosis in HCC patients [23–27]. However, the detailed molecular mechanism of how SMYD3 promotes HCC tumorigenesis and metastasis needs further investigation.

Transcriptional regulation through chromatin compaction and decompaction is mediated by various chromatin-remodeling complexes, such as nucleosome remodeling and histone deacetylation (NuRD) complexes [28]. The subunits of the NuRD complex include CHD3/4/5, HDAC1/2, MBD2/3, RBBP4/7, MTA1/2/3, and GATADA/B. Among these, HDAC1/2 contains histone deacetylase activity, and CHD3/4/5 is attributed to chromatin remodeling activity [29, 30]. The NuRD complex acts as a transcriptional corepressor and is recruited by sequence-specific transcription factors to induce robust gene silencing [31]. It is a highly conserved and ubiquitously expressed multi-subunit complex and plays an essential role during normal development as well as in a broad range of human diseases, including cancer [28, 32].

In this study, we conducted RNA-seq analysis on 5 pairs of HCC and adjacent noncancerous tissues and found that SMYD3 was highly expressed in tumor tissue. Expression of SMYD3 was positively associated with the clinical grade of HCC. We found that SMYD3 represses a cohort of genes, some of which are critically involved in cell proliferation and invasion through coordinating with the NuRD (MTA1/2) complex. We revealed that SMYD3 promotes cell proliferation and metastasis in vitro and in vivo. Our data revealed that SMYD3 regulated transcriptional repression by selectively associating with the NuRD (MTA1/2) complex to promote tumorigenesis and metastasis, identifying a previously unrecognized mechanism for driving tumorigenesis in HCC.

## Results

### SMYD3 expression is upregulated in HCC tissues

**Fig. 1:**
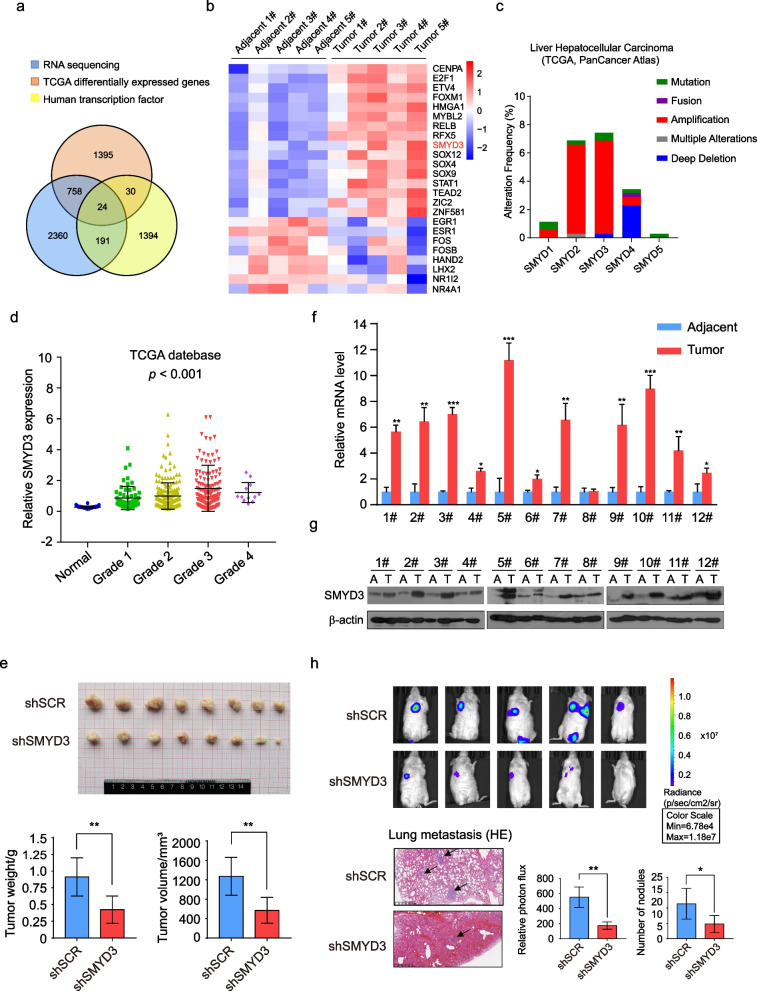
SMYD3 expression is upregulated in HCC tissues. **a**, **b** We investigated the transcriptomes in 5 pairs of HCC and adjacent noncancerous tissues using a high-throughput RNA deep sequencing approach (RNA-seq). Then, the results were integrated with the differentially expressed genes of HCC and adjacent tissues in the TCGA database (2207 genes) and The Human Transcription Factors database (1639 genes), and 24 differentially expressed regulatory factors were identified. **c** The alteration frequency of the SMYD family in HCC from TCGA database. **d** Analysis of clinical data from TCGA database for expression of SMYD3 by disease stage progression. **e** LM3 cells stably expressing SMYD3 shRNA or shSCR were transplanted into athymic mice. The tumor was dissected at 4 weeks after injection, and the images were presented. The average tumor mass and weight of each group are shown. **P < 0.01 (two-tailed unpaired *t*-test). **f** Real-time PCR of SMYD3 in 12 pairs of HCC and adjacent noncancerous tissues. Expression of SMYD3 was measured using qPCR. In addition, mRNA levels were normalized to β-actin. Each bar represents the mean ± s.d. for triplicate experiments (*P < 0.05, **P < 0.01, ***P < 0.001). **g** Western blotting analysis of the protein levels of SMYD3 in 12 pairs of HCC and adjacent noncancerous tissues. **h** LM3-Luc cells infected with lentiviruses carrying the indicated shRNAs were injected intravenously through the tail vein of 6-week-old female SCID mice (*n* = 5). Lung metastasis was quantified using bioluminescence imaging after 4 weeks

To explore the key regulatory factors affecting the tumorigenesis and metastasis of HCC, we investigated the transcriptomes in 5 pairs of HCC and adjacent normal tissues using a high-throughput RNA deep sequencing approach (RNA-seq), and 3333 differentially expressed genes were identified (Additional file 2). Then, the RNA-seq results were integrated with the differentially expressed genes of HCC and adjacent tissues in the TCGA database (2207 genes) and The Human Transcription Factors database (1639 genes) [33], and 24 differentially expressed regulatory factors were found (Fig. 1a). These 24 genes are shown in Fig. 1b, and among them, we were interested in SMYD3. SMYD3, which belongs to the SMYD family, could catalyze histone and nonhistone methylation and is a potential transcription factor. Analysis of the TCGA database using cBioportal (http://www.cbioportal.org/) [34] indicated that SMYD3 mutation frequencies were in nearly 8% of HCC cases, and these alterations were caused by gene amplification, mutation, and deletion, of which, gene amplification accounted for the majority (Fig. 1c). The results from TCGA database also indicated increased expression levels of SMYD3 in tumor tissues compared to noncancerous tissues, and its expression tended to be positively correlated with disease stage progression (Fig. 1d). In addition, we investigated the role of SMYD3 in tumor development and progression in vivo by subcutaneously implanting LM3 cells that had been engineered to stably express SMYD3 shRNA or control scrambled shRNA into athymic BALB/c mice. Growth of the implanted tumors was monitored by measuring tumor size every 3 days over a period of 4 weeks. The results revealed that tumor growth was substantially decreased in response to SMYD3 knockdown. The average tumor weight/volume of mice in the SMYD3 interference group was 46.4%/44.9% of that in the control group (Fig. 1e). To confirm this finding, we collected 12 pairs of HCC and adjacent noncancerous tissues and found that the mRNA/protein level of SMYD3 was upregulated in the tumor tissues (Fig. 1f. g). To assess the function of SMYD3 in tumor metastasis, LM3 cells stably expressing firefly luciferase were infected with lentiviruses carrying shSMYD3 or shSCR. Then, the cells were intravenously injected into immunocompromised severe combined immunodeficient (SCID) male mice (*n* = 5). Metastatic tumors were measured using quantitative bioluminescence imaging after 4 weeks using an IVIS imaging system (Xenogen). We found that SMYD3 deficiency significantly reduced the number of tumor nodules and HCC cell lung metastasis in vivo (Fig. 1h). These results indicated that SMYD3 is highly expressed in tumor tissues and may play an important role in tumor proliferation and metastasis.

### SMYD3 accelerates HCC proliferation and invasion

**Fig. 2:**
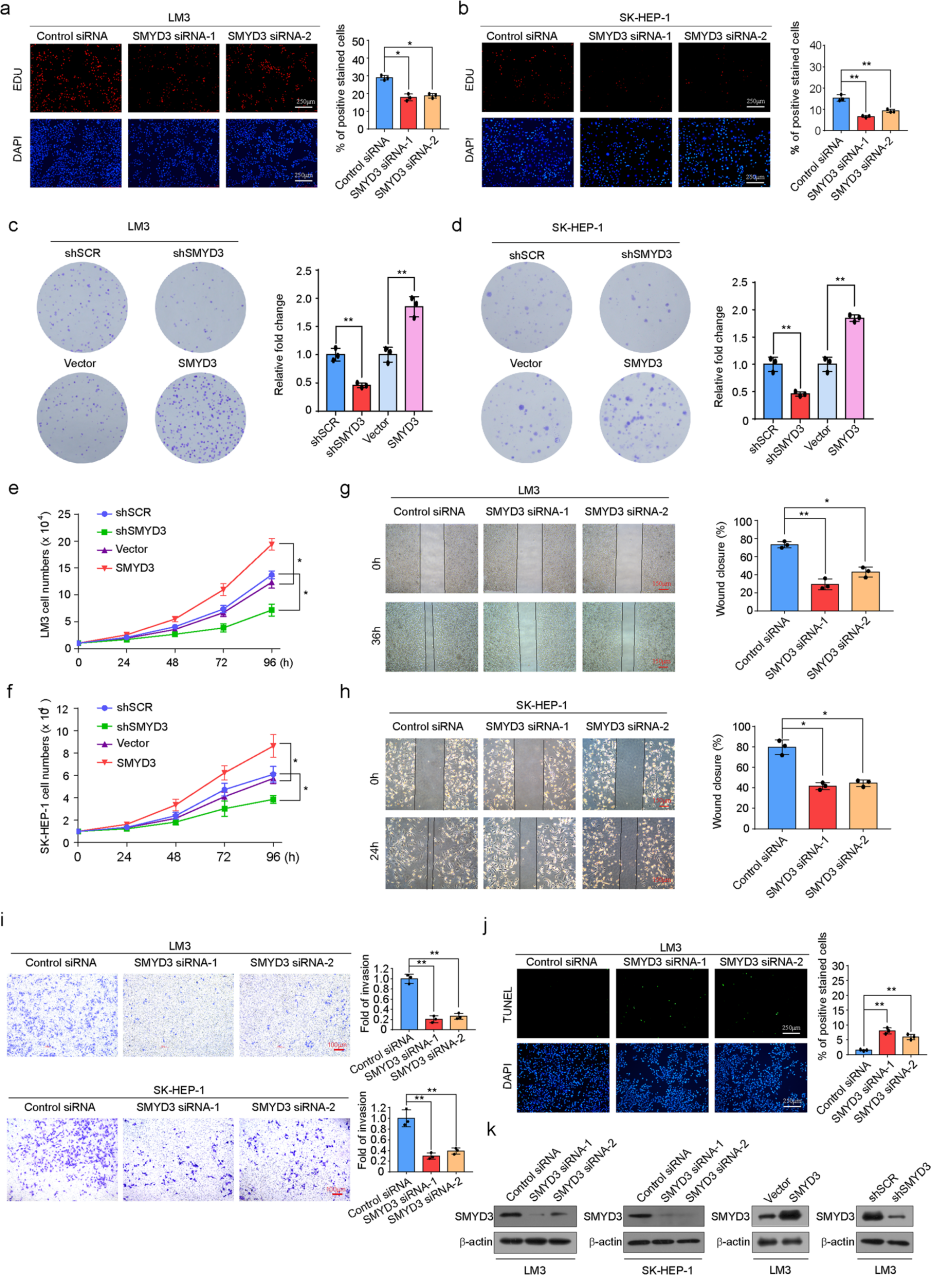
Knockdown of SMYD3 inhibits HCC carcinogenesis and invasion. **a**, **b** SMYD3 knockdown decreases the cellular proliferation of LM3 cells and SK-HEP-1 cells. EdU incorporation assays were performed using a fluorescence method. Representative images and statistical analyses are shown. **c**, **d** Colony formation assays of LM3 and SK-HEP-1 cells stably infected with SMYD3 shRNA, shSCR, Flag-SMYD3 or vector were maintained in culture media for 10 days prior to staining with crystal violet. Representative images and statistical analyses are shown. **e**, **f** LM3 and SK-HEP-1 cells stably expressing Flag-SMYD3, vector or the indicated lentivirus-delivered constructs (shSCR or shSMYD3) were subjected to growth curve analysis by counting the numbers of living cells. **g**, **h** Wound-healing assays of SMYD3-depleted LM3 and SK-HEP-1 cells were performed. Representative images and statistical analyses are shown, *P < 0.05, **P < 0.01 (two-tailed *t*-test). **i** Transwell invasion assays of LM3 and SK-HEP-1 cells were performed following transfection with the indicated specific siRNA. The invaded cells were stained and counted. The images represent one field under microscopy in each group, and statistical analyses are shown. **P < 0.01 (two-tailed *t*-test). **j** TUNEL assays of LM3 cells following transfection with the indicated specific siRNA were performed. The apoptotic cells were stained and quantified. The images represent one field under microscopy in each group, and statistical analyses are shown. **P < 0.01 (two-tailed *t*-test). **k** The interference or overexpression efficiency of SMYD3 in LM3 and SK-HEP-1 cells is shown

To investigate the function of SMYD3 in HCC, we first used an enhanced BrdU (EdU) incorporation assay and found that SMYD3 depletion was associated with a decreased mitotic rate compared to controls in both LM3 and SK-HEP-1 cells (Fig. 2a, b). Using colony formation assays, we found that in LM3 cells, SMYD3 knockdown was associated with a marked decrease in colony number, whereas SMYD3 overexpression was associated with a significant increase in colony number in LM3 cells (Fig. 2c). Consistent results were obtained in SK-HEP-1 cells (Fig. 2d). Growth curve assays revealed that SMYD3 knockdown significantly reduced cell growth and that SMYD3 overexpression markedly accelerated cell growth in both LM3 cells (Fig. 2e) and SK-HEP-1 cells (Fig. 2f). In addition, the impact of loss of function of SMYD3 on the migration and invasion of these cells was assessed using wound-healing assays and transwell invasion assays. In the wound-healing assays, the remaining open distance of cells with altered SMYD3 levels was different from that of the control group 24 or 36 h after migration. SMYD3 knockdown was associated with a decreased migration rate in LM3 (Fig. 2g) and SK-HEP-1 (Fig. 2h). The results from transwell invasion assays in two HCC cell lines (LM3 and SK-HEP-1) revealed significantly decreased invasiveness in response to SMYD3 knockdown (Fig. 2i). Furthermore, using a TUNEL assay, we found that SMYD3 knockdown elevated the apoptosis rate of LM3 cells (Fig. 2j). Quality testing of the interference efficiency of SMYD3 and SMYD3 overexpression in LM3 and SK-HEP-1 cells is shown in Fig. 2k. These findings support the notion that SMYD3 accelerates HCC cell proliferation and invasion and inhibits apoptosis.

### Proteomic analysis of the SMYD3 interactomes

**Fig. 3:**
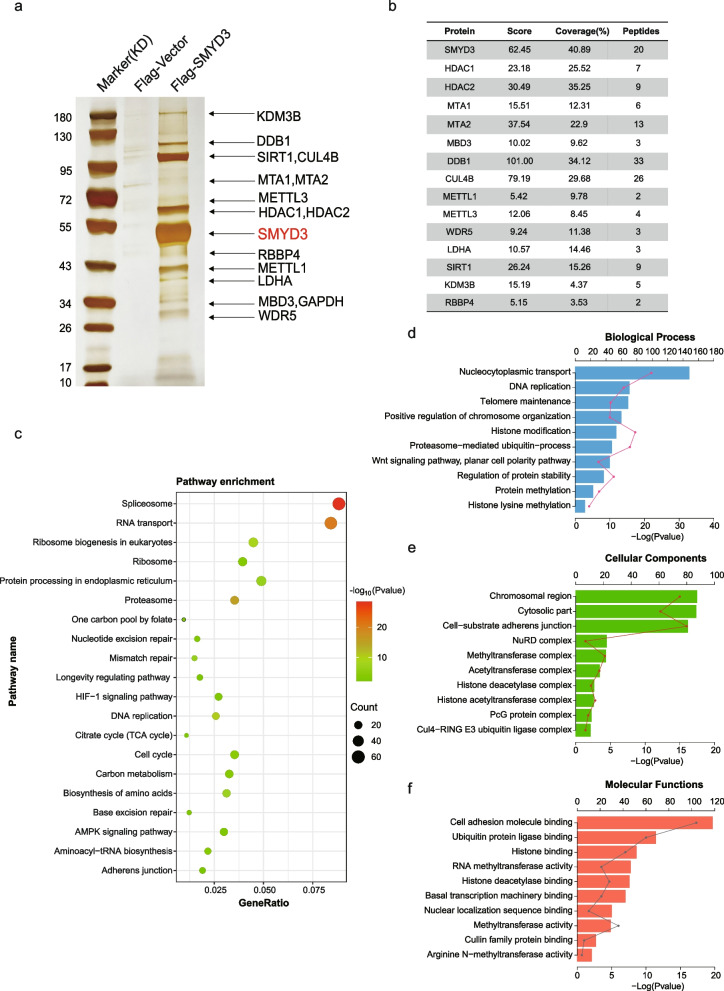
Proteomic analysis of the SMYD3 interactomes. **a** Immunoaffinity purification of SMYD3-containing protein complex. Cellular extracts from LM3 cells stably expressing FLAG-Vector or FLAG-SMYD3 were immunopurified using anti-FLAG affinity gel and eluted with the FLAG peptide. These eluents were resolved using SDS–PAGE and silver stained. **b** Score and peptide coverage of the indicated proteins. **c** KEGG pathway analysis of the SMYD3-interacting proteins. **d**–**f** GO analysis of putative functional processes associated with SMYD3-interacting proteins

In an effort to better understanding the mechanistic role of SMYD3, affinity purification and mass spectrometry assays were used to identify proteins associated with SMYD3 in vivo. In these experiments, FLAG-tagged SMYD3 or an empty vector was stably expressed in LM3 cells. Cellular extracts were prepared and subjected to affinity purification using an anti-FLAG affinity gel. Immunocomplexed proteins were separated using SDS–PAGE and silver stained (Fig. 3a, b). Immunoprecipitated proteins in specific bands in comparison to the vector were gel extracted, trypsin digested, and identified using liquid chromatography tandem mass spectrometry. Surprisingly, > 700 unique proteins with a protein score equal to or higher than two were identified (Additional file 3). To identify putative functional processes associated with SMYD3-interacting proteins, we next performed KEGG pathway [35] enrichment analysis and Gene Ontology (GO) analysis [36] for these interacting proteins. KEGG pathways analysis showed that SMYD3-interacting proteins are associated with regulation of many vital biological processes and activities (Fig. 3c). The top-ranked categories of biological process (BP) analysis were nucleocytoplasmic transport, DNA replication, telomere maintenance, positive regulation of chromosome organization, histone modification, and so on, suggesting that SMYD3 is related to gene transcriptional regulation and expression (Fig. 3d). In addition, cellular component (CC) analysis showed that SMYD3-interacting proteins were related to chromosomal region, cytosolic part, cell-substrate adherens junction, NuRD complex, methyltransferase complex, PcG protein complex, Cul4-RING E3 ubiquitin ligase complex, and so on, implying that SMYD3 is likely to participate in cell adhesion and invasion (Fig. 3e). Molecular function (MF) analysis identified many predominant themes, including cell adhesion molecule binding, ubiquitin protein ligase binding, histone binding, and RNA methyltransferase activity. This indicates that SMYD3 may be involved in epigenetic regulation, such as DNA/RNA or histone modification and chromatin remodeling (Fig. 3f). To further research the functional relationship between SMYD3-interacting proteins and specific functional complexes, a PPI network of the identified proteins was constructed using the STRING online database (http://string-db.org) [37]. We are interested in the role of SMDY3 in epigenetic regulation, so we selected 76 proteins involved in epigenetic regulation among more than 700 SMYD3 interacting proteins and the PPI analysis revealed multiple SMYD3-associated complexes, which suggest previously unknown functions of SMYD3 (Additional file 4: Fig. S1). Notably, the PPI analysis identified nearly every component of the NuRD complex, which is important in histone deacetylation and chromatin remodeling.

### SMYD3 physically associates with the NuRD complex

**Fig. 4:**
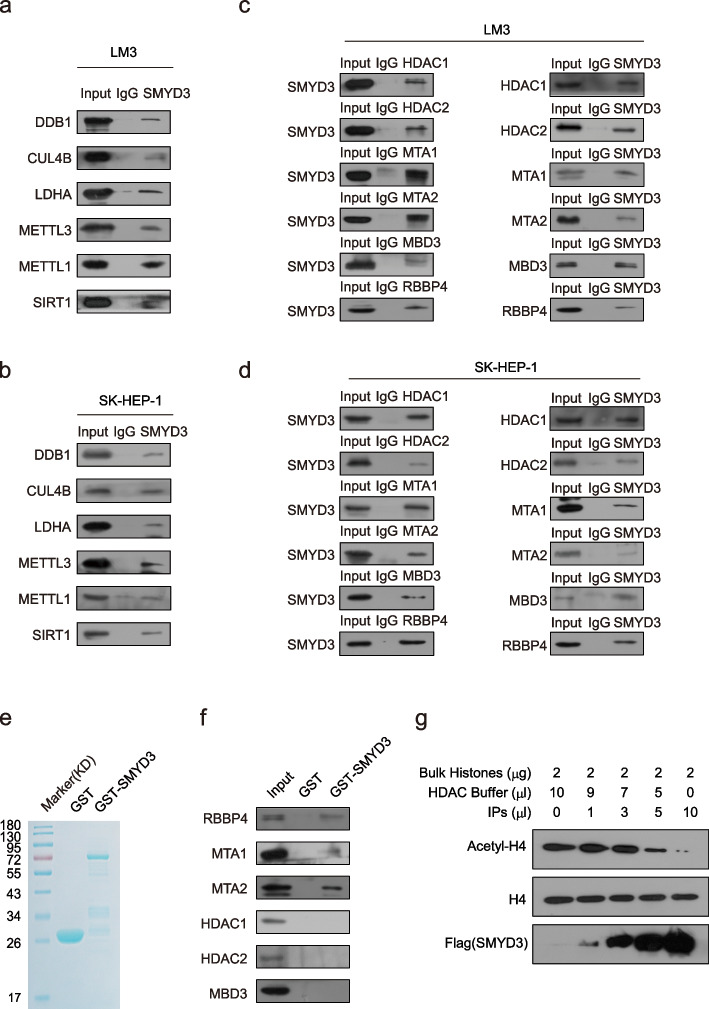
SMYD3 physically associates with the NuRD complex. **a**, **b** Whole cell lysates from LM3 and SK-HEP-1 cells were prepared, and co-IP was performed using antibodies against SMYD3. Immunocomplexes were then IB tested using antibodies against the indicated proteins. IgG served as the negative control. **c**, **d** Association of SMYD3 with the NuRD complex in LM3 and SK-HEP-1 cells. Whole cell lysates were immunoprecipitated using antibodies against the indicated proteins. Immunocomplexes were then IB tested using antibodies against the indicated proteins. **e** Coomassie blue staining of GST-SMYD3 purified from bacterial. **f** GST pull-down experiments using bacterially expressed GST-fused proteins and in vitro transcribed/translated components of the NuRD complex. **g** Cellular extracts were obtained from LM3 cells stably expressing FLAG-SMYD3 and were immunoprecipitated with anti-FLAG antibody. The immunoprecipitates (IPs) were incubated with bulk histones and histone deacetylation (HDAC) assay buffer. The reaction mixtures were analyzed by western blotting using antibodies against the indicated histone marks or proteins

To further confirm the in vivo interaction between SMYD3 and its interacting proteins, total proteins from LM3 and SK-HEP-1 cells were extracted, and co-IP was performed using antibodies detecting endogenous proteins. IP with antibodies against SMYD3 followed by IB with antibodies against DDB1, CUL4B, LDHA, METTL3, METTL1, and SIRT1 demonstrated that these proteins were efficiently coimmunoprecipitated with SMYD3 in LM3 and SK-HEP-1 cells (Fig. 4a, b). As previously mentioned, almost every component of the NuRD complex was identified in the mass spectrometry results. Therefore, we conducted IP using antibodies against SMYD3 followed by IB with antibodies against components of the NuRD complex and found that they were efficiently coimmunoprecipitated with SMYD3 in LM3 cells. In turn, IP with antibodies against representative components of the NuRD complex and IB with antibodies against SMYD3 reinforced the finding that SMYD3 was efficiently coimmunoprecipitated with the NuRD complex components (Fig. 4c). The same results were obtained in SK-HEP-1 cells (Fig. 4d). In addition, we explored the molecular basis for the interaction between SMYD3 and the NuRD complex by using GST pull-down assays. We used GST-fused SMYD3 (Fig. 4e) and in vitro transcribed/translated components of the NuRD complex. These experiments revealed that SMYD3 directly interacts with MTA1/2 and RBBP4 (Fig. 4f). To further investigate the physical association and to examine the functional connection between SMYD3 and the NuRD complex, enzymatic activity assay was performed to investigate whether SMYD3 associated with an HDAC enzymatic activity. The immunoprecipitates (IPs) were first incubated with bulk histones isolated from LM3 cells, and the levels of acetylated histones in the reactions were then analyzed by western blotting. Notably, the SMYD3-containing complex indeed possessed an enzymatic activity that led to a significant decrease in the acetylation level of H4 (Fig. 4g). The results indicate that the SMYD3-containing complex possesses histone deacetylation activities by interacting with the NuRD complex.

### Transcription target analysis for the SMYD3/NuRD complex

**Fig. 5:**
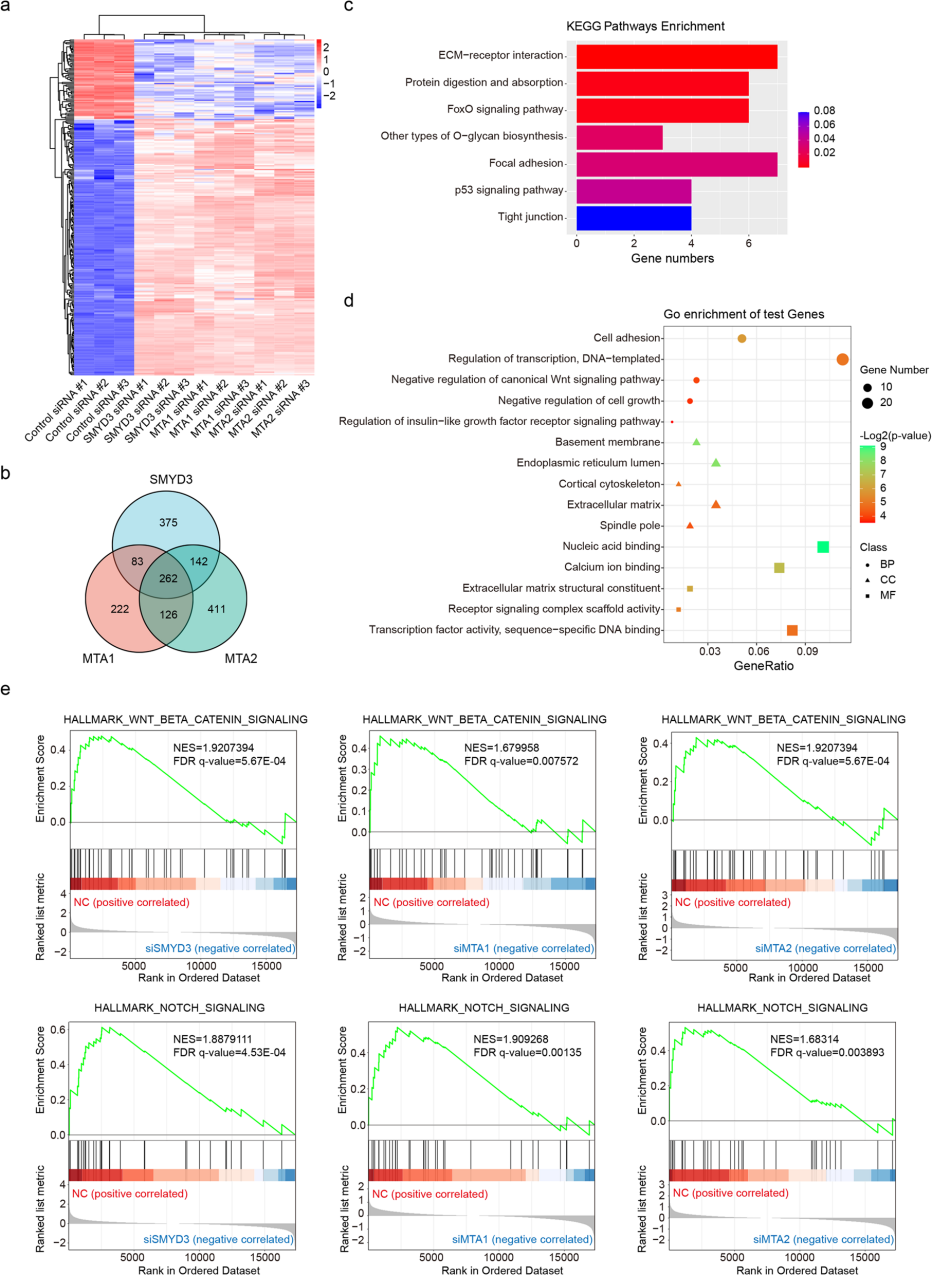
Transcriptional target analysis for the SMYD3/NuRD complex. **a** LM3 cells were transfected with control siRNA, SMYD3 siRNA, MTA1 siRNA, or MTA2 siRNA followed by RNA extraction and deep sequencing. Three independent samples were used for the RNA-seq analysis. mRNA expression data were clustered using Cluster 3.0 software. **b** Intersection of SMYD3-, MTA1-, and MTA2-regulated transcriptomes revealed 262 coregulated genes with a fold change ≥ 2. **c**, **d** KEGG pathways enrichment analysis and GO analysis of the co-target genes of SMYD3, MTA1, and MTA2 were performed to further explore the function of the SMYD3/NuRD(MTA1/MTA2) complex. **e** GSEA results showing enrichment gene signatures related to Wnt β-catenin signaling pathway and Notch signaling pathway in LM3 cells in response to SMYD3, MTA1, or MTA2 knockdown

To further investigate and understand the biological significance of the interaction between SMYD3 and the NuRD (MTA1/MTA2) complex, we investigated the transcriptomes of SMYD3-, MTA1-, or MTA2-deficient LM3 cells using a high-throughput RNA deep sequencing approach (RNA-seq). Total RNA was extracted from LM3 cells transfected with control shRNA (shSCR) or shRNA-targeting SMYD3, MTA1, or MTA2. RNA-seq analysis identified 862, 693, and 941 genes whose expression was altered in response to SMYD3, MTA1, and MTA2 depletion, respectively (Fig. 5a, Additional file 5). Cross-analysis of the transcriptomes from SMYD3-, MTA1-, and MTA2-deficient cells identified 262 genes whose expression was altered in SMYD3-, MTA1-, and MTA2-depleted cells. These target genes were coregulated by SMYD3, MTA1, and MTA2 (Fig. 5b). To identify the putative functional processes associated with the targets that were coregulated by SMYD3, MTA1, and MTA2, we performed KEGG pathway enrichment analysis and classified the genes into various cellular signaling pathways, including ECM-receptor interaction, protein digestion and absorption, FoxO signaling pathway, focal adhesion, and p53 signaling pathway, which are all critically involved in cell growth, migration, and invasion (Fig. 5c). We also performed Gene Ontology (GO) analysis. The top-ranked categories of the biological process (BP) analysis included negative regulation of cell growth, negative regulation of canonical Wnt signaling pathway, regulation of insulin-like growth factor receptor signaling pathway, cell adhesion, and regulation of transcription. (Fig. 5d), suggesting that the association of SMYD3, MTA1, and MTA2 may be related to proliferation, epithelial-mesenchymal transition, and gene transcription regulation. Gene set enrichment analyses (GSEAs) [38] revealed enrichment in the Wnt β-catenin signaling pathway and Notch signaling pathway in response to SMYD3, MTA1, or MTA2 knockdown (Fig. 5e).

**Fig. 6:**
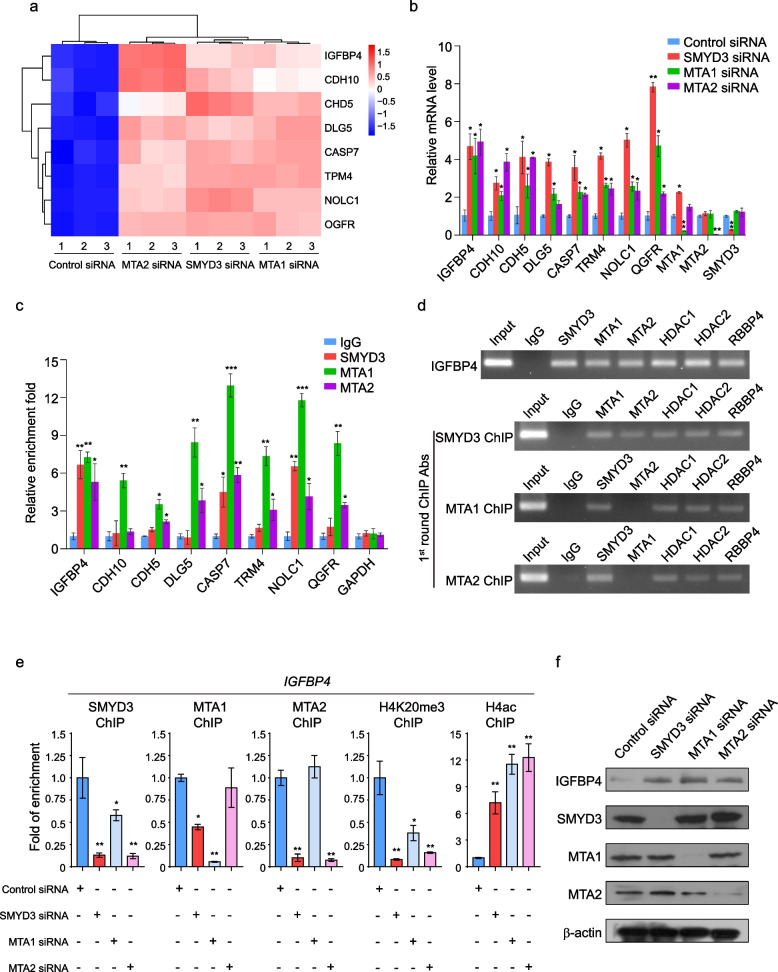
Validation of the transcriptional regulation of SMYD3, MTA1, and MTA2 on target genes. **a** A heatmap presents the selected target genes that were coregulated by SMYD3, MTA1, and MTA2 in the RNA-seq analysis. **b** LM3 cells were infected with control siRNA, SMYD3 siRNA, MTA1 siRNA, or MTA2 siRNA. RT–PCR analyses of partial transcriptional target expression in LM3 cells infected with the indicated siRNAs are shown. **c** qChIP analysis of the selected promoters was performed using antibodies against IgG, SMYD3, MTA1, and MTA2. Results are represented as the fold change over IgG with glyceraldehyde 3-phosphate dehydrogenase as a negative control. Error bars represent the mean ± SD of three independent experiments (*P < 0.05, **P < 0.01, ***P < 0.001 and two-tailed unpaired *t*-test). **d** ChIP/Re-ChIP analysis of the IGFBP4 promoter was performed using antibodies against SMYD3, MTA1, MTA2, HDAC1, HDAC2, and RBBP4. IgG served as a negative control. **e** qChIP analysis of the recruitment of the indicated proteins on the IGFBP4 promoter in LM3 cells after infection with control siRNA, SMYD3 siRNA, MTA1 siRNA, or MTA2 siRNA. Purified rabbit IgG was used as a negative control. Error bars represent the mean ± SD of three independent experiments. *P < 0.05 and **P < 0.01 (two-tailed *t*-test). **f** Western blot analyses of IGFBP4 protein expression in LM3 cells infected with the indicated siRNAs are shown

To validate the RNA-seq analysis, we chose 8 representative genes and validated their expression in LM3 cells using quantitative real-time PCR (qPCR). Figure 6a shows the 8 representative genes whose expression were altered in RNA-seq data. qPCR results indicated that mRNA levels of *IGFBP4*, *CDH10*, *CHD5*, *DLG5*, *CASP7*, *TPM4*, *NOLC1*, and *OGFR* were increased upon the knockdown of either SMYD3 or MTA1/MTA2 compared to control siRNA (Fig. 6b). Then, we examined whether SMYD3 and MTA1/MTA2 regulated target gene expression by binding to their promoters. qChIP assays in LM3 cells using antibodies against SMYD3, MTA1, MTA2, or control IgG revealed that SMYD3 and MTA1/MTA2 co-occupied the promoters of *IGFBP4*, *CASP7*, and *NOLC1*. The ChIP results were quantitated using qPCR (Fig. 6c). To further test our proposition that SMYD3 and MTA1/MTA2 function in the same protein complex at target promoters, we performed sequential ChIP/Re-ChIP using antibodies against SMYD3, MTA1, MTA2, HDAC1, HDAC2, and RBBP4 for IGFBP4. The results demonstrated that the *IGFBP4* promoter that was immunoprecipitated with antibodies against SMYD3 could be reimmunoprecipitated with antibodies against MTA1, MTA2, HDAC1, HDAC2, and RBBP4. Similar results were obtained when the initial ChIP was performed using antibodies against MTA1 or MTA2 (Fig. 6d). These results support the conclusion that SMYD3 and MTA1 or MTA2 occupy co-target gene promoters as functionally collaborative protein complexes. qChIP analyses showed that SMYD3 knockdown led to a significant reduction in the binding of MTA1 or MTA2 to the promoters of IGFBP4, suggesting that SMYD3 could specifically affect NuRD complex to the target gene promoters. Notably, knockdown of either SMYD3 or MTA1 or MTA2 led to a significant decrease in H4K20me3 and a marked increase in H4Ac at the IGFBP4 promoter (Fig. 6e). When we interfered with SMYD3, MTA1, or MTA2 using the corresponding siRNA, protein levels of IGFBP4 were remarkably increased (Fig. 6f).

**Fig. 7:**
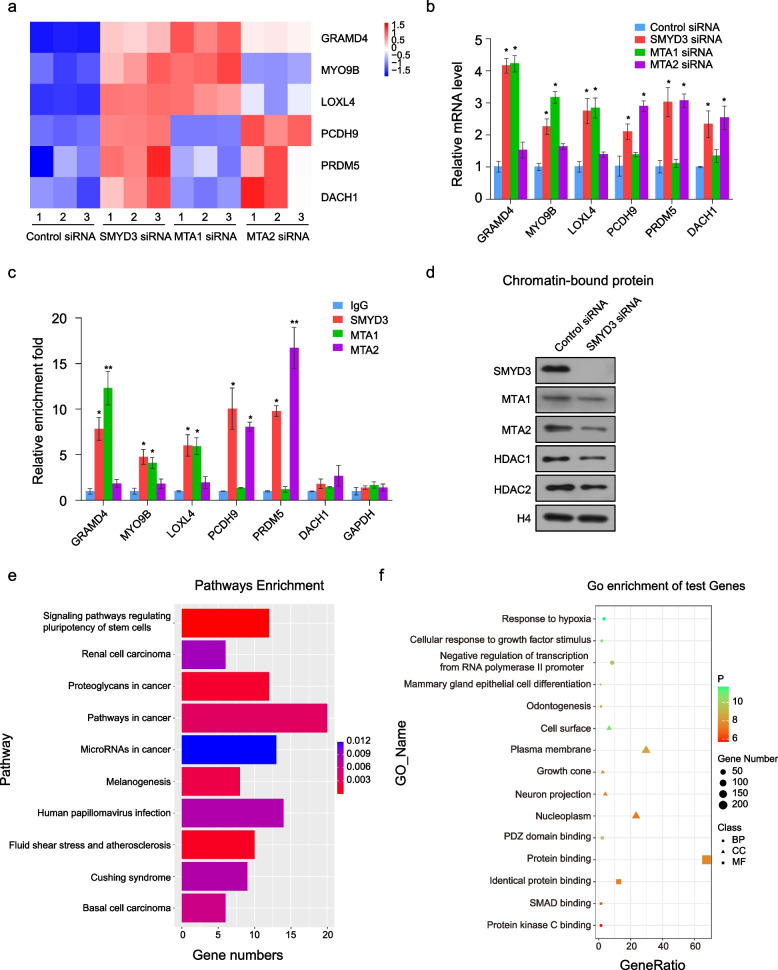
Identification the different target genes between SMYD3/NuRD (MTA1) complex and SMYD3/NuRD (MTA2) complex. **a** A heatmap presents the selected target genes that were coregulated by SMYD3 and MTA1 (or MTA2) in the RNA-seq analysis. **b** LM3 cells were infected with control siRNA, SMYD3 siRNA, MTA1 siRNA, or MTA2 siRNA. RT–PCR analyses of partial transcriptional target expression in LM3 cells infected with the indicated siRNAs are shown. **c** qChIP analysis of the selected promoters was performed using antibodies against IgG, SMYD3, MTA1, and MTA2. Results are represented as the fold change over IgG with glyceraldehyde 3-phosphate dehydrogenase as a negative control. **d** The chromatin-bound proteins in Control siRNA and SMYD3 siRNA transfected LM3 cells were extracted and subjected to analysis to determine the indicated protein levels. Total histone H4 served as a loading control. **e**, **f** KEGG pathways enrichment analysis and GO analysis of the target genes exclusively regulated by SMYD3

To further investigate the target genes specifically regulated by the SMYD3/NuRD (MTA1) complex or the SMYD3/NuRD (MTA2) complex, we next analyzed RNA-seq result and found that there are 83 target genes specifically regulated by SMYD3 and MTA1, and 142 target genes specifically regulated by SMYD3 and MTA2. To validate the RNA-seq analysis, we chose 6 representative genes which were negatively regulated by SMYD3/MTA1 or SMYD3/MTA2 (Fig. 7a), and validated their expression in LM3 cells using quantitative real-time PCR (qPCR). The results indicate that the mRNA levels of *GRAMD4*, *MYO9B*, and *LOXL4* increased upon the knockdown of either SMYD3 or MTA1, but no significant change upon the knockdown of MTA2. Similarly, the mRNA levels of *PCDH9*, *PRDM5*, and *DACH1* increased upon the knockdown of either SMYD3 or MTA2, but no significant change upon the knockdown of MTA1 (Fig. 7b). qChIP assays with antibodies against SMYD3, MTA1, MTA2, or control IgG revealed that SMYD3 and MTA1 specifically co-occupied the promoters of *GRAMD4*, *MYO9B*, and *LOXL4*. SMYD3 and MTA2 specifically co-occupied the promoters of *PCDH9* and *PRDM5*, but not *DACH1* (Fig. 7c). To further test whether SMYD3 affects the NuRD complex binding to chromatin, chromatin-binding proteins were extracted from the SMYD3 knockdown LM3 cells. The results showed that as compared to control, the amount of chromatin-bound MTA1, MTA2, HDAC1, and HDAC2 was slightly decreased with SMYD3 knockdown (Fig. 7d). In addition, knockdown of HDAC1 or HDAC2 led to an increased expression of global H4 acetylation level in LM3 cells (Additional file 6: Fig. S2a). To further investigate the function of SMYD3 independent of NuRD complex, we performed KEGG pathway analysis (Fig. 7e) and GO analysis (Fig. 7f) on 375 target genes exclusively regulated by SMYD3. The results indicated that SMYD3 was associated with regulation of many vital cell processes and activities, such as signaling pathways regulating pluripotency of stem cells and response to hypoxia, which was different from the signaling pathway regulated by coordinating with the NuRD complex, suggesting that SMYD3 is also involved in vital biological processes that are independent of the NuRD complex.

**Fig. 8:**
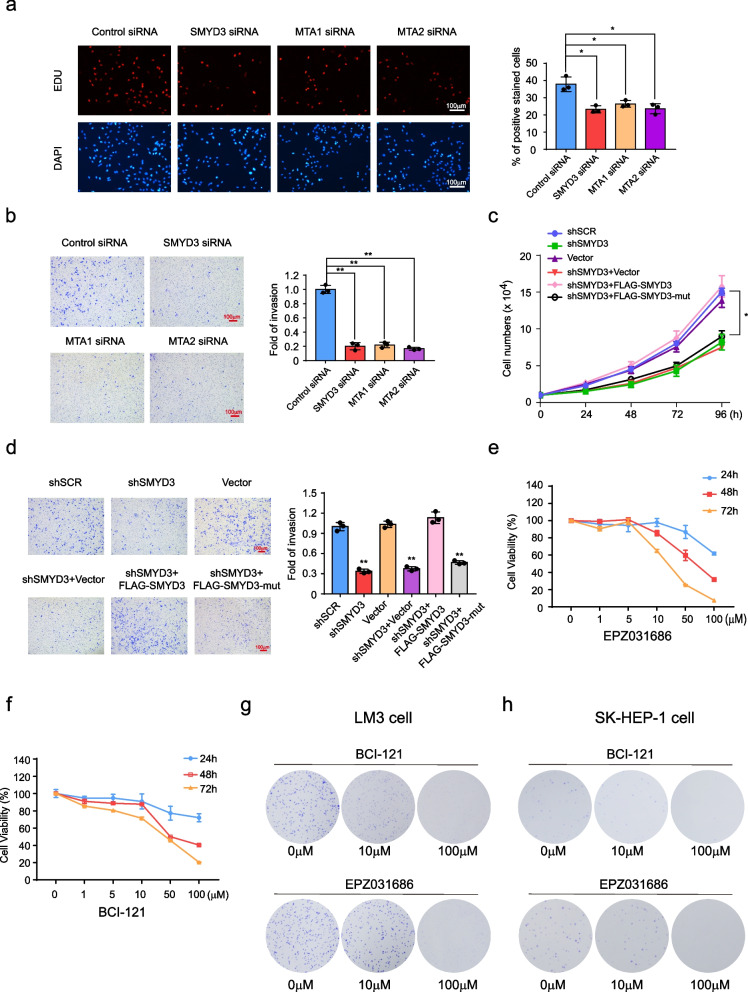
Inhibitors of SMYD3 effectively inhibit the proliferation of HCC cells. **a** MTA1, MTA2 knockdown decrease the cellular proliferation of LM3 cells. EdU incorporation assays were performed using a fluorescence method. Representative images and statistical analyses are shown. **b** Transwell invasion assays of LM3 cells were performed following transfection with the indicated specific siRNA. The invaded cells were stained and counted. The images represent one field under microscopy in each group, and statistical analyses are shown. **P < 0.01 (two-tailed *t*-test). **c** LM3 cells transfected with the indicated plasmid or/and the indicated lentivirus-delivered constructs (shSCR or shSMYD3) were subjected to growth curve analysis by counting the numbers of living cells. **d** Transwell invasion assays of LM3 cells were performed following transfection with the indicated lentivirus-delivered constructs (shSCR or shSMYD3) or/and the indicated plasmid. The invaded cells were stained and counted. The images represent one field under microscopy in each group, and statistical analyses are shown. **P < 0.01 (two-tailed t-test). **e**, **f** MTT assays were performed to analyze the viability of LM3 cells treated with an inhibitor of SMYD3 (EPZ031686 or BCI121) at different times and concentrations. **g**, **h** Colony formation assays were performed to analyze the cell proliferation ability of LM3 and SK-HEP-1 cells treated with EPZ031686 or BCI121

Based on our observations that SMYD3 and the NuRD (MTA1/MTA2) complex are physically and functionally associated, we next investigated if MTA1 or MTA2 have the same role of SMYD3 in tumor proliferation and invasion. We performed EdU assays and found that knockdown of MTA1 or MTA2 were associated with a decreased mitotic rate compared to the control, which have the same effects as SMYD3 knockdown (Fig. 8a). In addition, using transwell invasion assays, we found that knockdown of MTA1 or MTA2 was associated with a marked reduction in cell invasion, consistent with the effects as SMYD3 knockdown (Fig. 8b). To assess whether the documented phenotypic effects were due to SMYD3 methyltransferase activity, we constructed catalytic mutant SMYD3 vector (SMYD3 ΔSET). Using growth curve assays and transwell invasion assays, we found that the decreased cell viability and invasiveness upon SMYD3 knockdown was retrieved by the overexpression of WT SMYD3, but could not be rescued by the ectopic expression of SMYD3 ΔSET (Fig. 8c, d), indicating that SMYD3 putative oncogenic function is associated with the methyltransferase activity. The western blots verified the efficacies of the siRNAs and plasmids used in these experiments (Additional file 6: Fig. S2b).

Moreover, we next performed rescue experiments. Using an EdU assay, we found that deletion of SMYD3 markedly decreased LM3 cell proliferation, while deletion of IGFBP4 had the opposite effect. In addition, suppression of cell proliferation caused by SMYD3 deletion could be rescued by reducing the expression of IGFBP4 (Additional file 7: Fig. S3a). In addition, results from transwell assays performed using LM3 cells demonstrated that deletion of SMYD3 caused a substantial reduction in cell invasion, and deletion of IGFBP4 significantly increased invasion. However, inhibition of the invasive potential of LM3 cells associated with deletion of SMYD3 was diminished by reducing the expression of IGFBP4 (Additional file 7: Fig. S3b). We confirmed the efficiency of the siRNAs using western blot analysis (Additional file 7: Fig. S3c).

### Inhibitors of SMYD3 effectively inhibit the proliferation of HCC cells

Because SMYD3 plays an important role in the progression of HCC, BCI-121 and EPZ031686, which are selective SMYD3 inhibitors, were used to treat HCC cells. We performed MTT assays and found that cell viability was markedly reduced after treatment with BCI-121 or EPZ031686; in addition, cell viability was time- and concentration-dependent. The same results were obtained in LM3 cells and SK-HEP-1 cells (Fig. 8e, f). In addition, using colony formation assays, we revealed that colony numbers were significantly reduced when LM3 cells and SK-HEP-1 cells were treated with BCI-121 or EPZ031686 (Fig. 8g, h). These findings confirmed that SMYD3 inhibition using BCI-121 or EPZ031686 effectively alleviated HCC cell viability and proliferative potential.

**Fig. 9:**
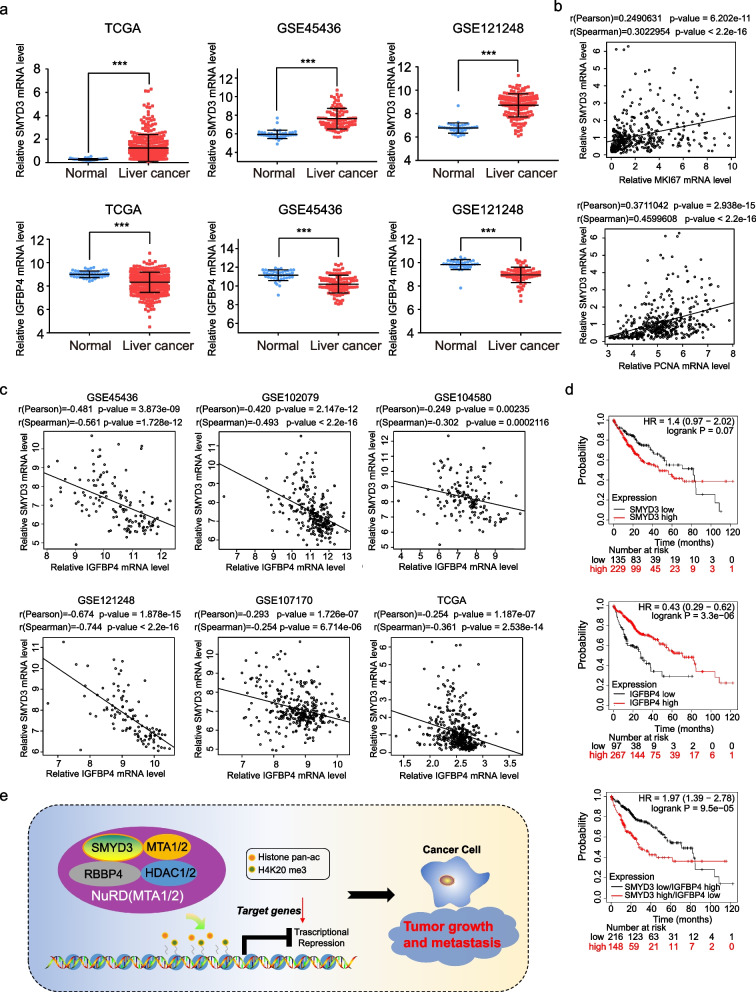
SMYD3 plays an important role in the clinicopathological significance of HCC. **a** Analysis of TCGA database and GSE database (GSE45436 and GSE121248) for the expression of SMYD3 using two-tailed unpaired *t*-test (***P < 0.001). **b** Analysis of the correlations between SMYD3 and PCNA or MKI67 in TCGA datasets. **c** Analysis of the correlations between SMYD3 and IGFBP4 in the public GSE45436, GSE102079, GSE104580, GSE121248, GSE107170, and TCGA datasets. **d** Kaplan–Meier survival analysis comparing high and low levels of SMYD3/IGFBP4 expression in liver cancer. **e** Graphic model as discussed in the text

We analyzed expression levels of SMYD3 and IGFBP4 in the TCGA database and in two published clinical datasets (GSE45436 and GSE121248) from the GEO database (Fig. 9a). The results revealed that expression of SMYD3 was upregulated, while IGFBP4 was downregulated in liver cancer tissues compared to noncancerous liver tissues. In addition, we found the expression of SMYD3 is significantly positively correlated with PCNA and MKI67 expression, which is a well-known marker of proliferation, in TCGA database (Fig. 9b). A reanalysis of the data sourced from published clinical datasets, such as GSE45436, GSE102079, GSE104580, GSE121248, GSE107170, and TCGA datasets, indicated that expression of SMYD3 is significantly negatively correlated with IGFBP4 expression, supporting our finding that IGFBP4 is transcriptionally inhibited by SMYD3 (Fig. 9c). In addition, Kaplan–Meier survival analysis (http://kmplot.com/analysis/) [39] revealed that lower expression of SMYD3 or overexpression of IGFBP4 was associated with improved overall survival in liver cancer patients. In addition, poor overall survival was associated with high expression of SMYD3 combined with low expression of IGFBP4 (Fig. 9d). In summary, these data support our overall hypothesis that SMYD3 cooperates with the NuRD (MTA1/MTA2) complex to inhibit expression of a series of tumor suppressor genes, leading to tumor progression (Fig. 9e).

## Discussion

An increasing number of studies have revealed that liver cancer is a multistep process that involves complicated interplays among genetics, epigenetics, and transcriptomic alterations [40]. Many transcription factors are associated with multiple hallmarks of cancers, and approximately 20% of all oncogenes that have been identified are transcription factors [41]. Moreover, histone modification is a crucial process in transcriptional regulation, while cancer‑associated epigenetic alterations play an important role in human carcinogenesis. Thus, it is essential to understand the underlying epigenetic molecular mechanisms in HCC. The current study provides evidence that SMYD3, a histone lysine methyltransferase and positive transcription factor, induces tumorigenesis by activating the expression of oncogenes. Michalis E. Sarris et al. provided solid evidence that SMYD3 can be recruited to its targets via multiple mechanisms, including interactions with trimethylated H3K4 tails, RNA Pol-II, or other sequence-specific binding proteins and required for liver and colon cancer development [23]. One recent study identified that SMYD3 bound to CDK2 and MMP2 promoter and increased H3K4me3 modification at the corresponding promoters to promote gene transcription [42]. In addition, SMYD3 could upregulate sphingosine-1-phosphate receptor 1 (S1PR1) promoter activity by methylating histone 3 (H3K4me3) and promotes HCC cell growth and migration in vitro and in vivo by upregulating S1PR1 expression [43]. However, the transcription repression mechanisms of SMYD3 in HCC still need to be characterized and studied. Here, our data indicate that SMYD3 interacts with multiple types of proteins involved in many vital signaling pathways and biological processes, such as ubiquitination, the NuRD complex, methyltransferase, and glucose metabolism. The protein interaction data further indicated the importance and multifunctionality of SMYD3. We demonstrated that SMYD3 associated with the NuRD (MTA1/2) complex to repress the expression of some tumor suppressor genes. The NuRD complex is composed of multiple enzymatic subunits and possesses ATPase, histone deacetylase, and histone demethylase enzymatic activities [44]. Metastasis-associated (MTA) family proteins, including MTA1, MTA2, and MTA3, are common components of the NuRD complex. Previous studies have indicated that MTA1 and MTA2 support tumor progression and EMT, while MTA3 inhibits EMT and cancer metastasis [45]. Our results revealed that SMYD3 specifically associates with the NuRD (MTA1/2) complex but not MTA3. SMYD3 directly interacts with MTA1 or MTA2 and represses transcription of the same target gene, *IGFBP4*. In contrast, knockdown of either MTA1 or MTA2 increased the expression of IGFBP4, suggesting that SMYD3 independently associates with NuRD (MTA1) or NuRD (MTA2) to regulate target gene expression and that MTA1 and MTA2 cannot compensate for each other. In addition, we also found that SMYD3/NuRD (MTA1) complex or SMYD3/NuRD (MTA2) complex could regulate specific target genes. For example, GRAMD4, MYO9B, and LOXL4 were negatively regulated by SMYD3/NuRD (MTA1) complex, while PCDH9, PRDM5, and DACH1 were specifically regulated by SMYD3/NuRD (MTA2) complex.

Among the findings of our MS results, we also observed that SMYD3 interacts with METTL3, LDHA, and WDR5. METTL3 is an m6A methyltransferase involved in all stages of the life cycle of RNA. LDHA is lactate dehydrogenase A, which catalyzes the conversion of l-lactate to pyruvate in the final step of anaerobic glycolysis. WDR5 interacts with methylated H3K4 to catalyze Lys4 trimethylation. Interestingly, SMYD3 also catalyzes dimethylation and trimethylation of H3K4, suggesting that SMYD3 plays an important role in transcriptional activation. Furthermore, SMYD3 also methylates the cytoplasmic portion of VEGFR1, increasing its kinase activity. SMYD3 was also shown to form a complex with RNA polymerase II and serves as a potential transcription factor [8]. Because SMYD3 coordinates with different epigenetic regulators, we suspect that SMYD3 can switch its role between transcriptional activation and repression by coordinating with different histone modification complexes.

The insulin-like growth factor (IGF) family consists of IGFs, IGF receptors, IGF binding proteins 1–6, and proteases of IGF binding proteins. IGFBP4 is the smallest member of the human IGFBPs and inhibits IGF-induced cellular growth. In addition, IGFBP-4 also regulates tumorigenesis through IGF-independent mechanisms. The liver is the primary source of circulating IGFBP-4, but this protein is expressed in adult tissues, including the liver, heart, brain, bone, ovary, muscle, prostate, and kidney [46]. Previous studies have shown that IGFBP4 is associated with cancers in lung, breast, liver, stomach, colon, prostate, bone, and nervous tissues [47, 48]. Loss of the IGFBP4 tumor suppressor drives H3K27me3-mediated epigenetic reprogramming in hepatic carcinogenesis [47]. EZH2 maintains the H3K27 methylome of the IGFBP4 promoter to repress its expression. Notably, we also demonstrated that SMYD3 selectively associates with NuRD (MTA1/2) complex to maintain the H4K20 methylation and deacetylation through epigenetic silencing of IGFBP4. Our results add to the understanding of the complexity of the regulatory mechanisms of IGFBP4.

## Conclusions

In summary, our study demonstrates that SMYD3 specifically associates with the NuRD (MTA1/2) complex to form a complex in liver cancer cells, providing new insights into how SMYD3 regulates transcription by coordinating with distinct partner protein complexes. Our results indicate that the SMYD3/NuRD complex is involved in liver cancer proliferation and metastasis. Our data provide a molecular basis for understanding the pathophysiological function of SMYD3, and its inhibition could have widespread application for liver cancer treatment.

## Methods

### Cell culture and transfection

LM3 cell line was purchased from the China Center for Type Culture Collection (Wuhan, China Cat. GDC0289), and SK-HEP-1 cell line was purchased from the Chinese Academy of Medical Sciences (Shanghai, China Cat. TCHu109). LM3 cells were cultured in DMEM medium (Gibco, Cat. C11995500BT), and SK-HEP-1 cells were cultured in MEM medium (Gibco, Cat. 12571–063). All media were supplemented with 10% fetal bovine serum (FBS) (BI, Cat. 04–001-1A), 100 units/ml penicillin, and 100 mg/ml streptomycin (Gibco, USA, Cat. 15140–122). Cells were cultured at 37 °C in a humidified incubator equilibrated with 5% CO_2_. Transfection was performed using TurboFect (Thermo Fisher Scientific, Cat. R0531) or Lipofectamine RNAiMAX reagent (Invitrogen, USA, Cat. 13778–150) according to the manufacturer’s instructions. For RNAi experiments, at least three independent siRNA sequences were tested for each gene, and the one with the best efficiency was used for subsequent experiments. All experiments were performed in triplicate and repeated at least three times.

### Lentiviral production and infection

Recombinant lentiviruses expressing shSMYD3 and shSCR were constructed by Shanghai GenePharma (Shanghai, China). Concentrated viruses with 8 μg/mL polybrene were used to infect 5 × 10^5^ cells in a 60-mm dish. Infected cells are specifically targeted for expression. shRNA sequences are listed in Additional file 1: Table S1.

### Antibodies and reagents

Anti-FLAG antibody was purchased from Sigma–Aldrich (Cat. F1804). Anti-SMYD3 (Cat. ab187149), anti-DDB1(Cat. ab109027), anti-METTL3 (Cat. ab195352), anti-HDAC1 (Cat. ab7028), anti-HDAC2 (Cat. ab7029), anti-MTA2 (Cat. ab8106), anti-Histone H4 (Cat. ab177840), and anti-MBD3 (Cat. ab157464) were purchased from Abcam. Anti-CUL4B (Cat. 12916–1-AP), anti-LDHA (Cat. 19987–1-AP), anti-METTL1 (Cat. 14994–1-AP), anti-IGFBP4 (Cat. 18500–1-AP), and anti-β-actin (Cat. 66009–1-lg) were purchased from Proteintech. Anti-MAT1 (Cat. D17G10) and anti-RBBP4 (Cat. 4633-S) were purchased from Cell Signaling Technology. Anti-SIRT1 antibody was purchased from Millipore (Cat. 07–131). Anti-Histone H4ac was purchased from ACTIVE MOTIF (Cat. 39243). Glutathione-Sepharose 4B beads were purchased from GE Healthcare Bio-Sciences (Cat. 17–0756-01). Dynabeads Protein G was obtained from Invitrogen by Thermo Fisher Scientific (Cat. 10004D), and the protease inhibitor mixture cocktail was from Roche Applied Science (Cat. 04 693 116 001). Inhibitors of SMYD3, including BCI-121 (Cat. HY-21972) and EPZ031686 (Cat. HY19324), were purchased from MedChemExpress (MCE). siRNAs were purchased from RiboBio (RiboBio Co. Ltd., Guangzhou, Guangdong). siRNA sequences are listed in Additional file 1: Table S1.

### Cloning

The FLAG–SMYD3 plasmid was generated by inserting the full-length SMYD3 sequence into the pCMV-Tag2B vector. FLAG–SMYD3-mut plasmid was generated by inserting the catalytic mutant SMYD3 sequence (ΔSET domain, deletion of 93-242aa) into the pCMV-Tag2B vector. The Glutathione *S*-transferase (GST)–NuRD plasmids were created by inserting full-length NuRD components into pGEX-4T-3 expression vectors as previously described [49].

### Immunopurification and mass spectrometry

Immunopurification and mass spectrometry analysis were performed as described previously [50]. In brief, LM3 cells were transfected with FLAG-Vector and FLAG-SMYD3 for 48 h. Lysates from LM3 cells expressing FLAG-Vector and FLAG-SMYD3 were incubated with ANTI-FLAG® M2 Magnetic Beads for about 1 h at room temperature with gentle mixing, according to the manufacturer’s instructions. The beads were then washed, followed by elution with FLAG peptides (0.2 mg/ml; Sigma–Aldrich, Cat. F4799). Fractions of the bed volume were collected and resolved on SDS–polyacrylamide gels, silver stained, and subjected to LC–MS/MS sequencing and data analysis. Data are available via ProteomeXchange with identifier PXD031971.

### Glutathione S-transferase (GST) pull-down experiments

GST fusion constructs were expressed in BL21 *Escherichia coli* cells, and crude bacterial lysates were prepared by sonication in cold PBS in the presence of a protease inhibitor mixture. In vitro transcription and translation experiments were performed using rabbit reticulocyte lysate (Promega, USA, Cat. L4950). In GST pull-down assays, ~ 10 μg of the appropriate GST fusion proteins was mixed with 5–8 μL of the in vitro transcribed/translated products and incubated in binding buffer (0.8% BSA in PBS with the protease inhibitor mixture). The binding reaction was then added to 30 μL of glutathione-Sepharose beads and mixed at 4 °C for 2 h. The beads were washed five times with binding buffer, resuspended in 30 μL of 2 × SDS–PAGE loading buffer, and resolved on 12% gels. Protein levels were detected by western blotting using specific antibodies.

### Immunoprecipitation

Cellular extracts were harvested and incubated with the appropriate primary antibody or normal mouse/rabbit immunoglobin G (IgG) at 4 °C overnight. Samples were mixed with protein A/G magnetic beads for 2 h at 4 °C, and following a wash, the beads underwent SDS–PAGE, followed by immunoblotting with a secondary antibody. Immunodetection was performed using enhanced chemiluminescence with an ECL System (Millipore, Cat. WBKLS0500) according to the manufacturer’s instructions.

### RNA sequencing

Total RNA was extracted from the tissues or cells using Trizol (Invitrogen, Carlsbad, CA, USA) according to manual instruction. Oligo(dT)-attached magnetic beads were used to purified mRNA. Purified mRNA was fragmented into small pieces with fragment buffer at appropriate temperature. Then first-strand cDNA was generated using random hexamer-primed reverse transcription, followed by a second-strand cDNA synthesis. Afterwards, A-Tailing Mix and RNA Index Adapters were added by incubating to end repair. The cDNA fragments obtained from previous step were amplified by PCR, and products were purified by Ampure XP Beads, then dissolved in EB solution. The product was validated on the Agilent Technologies 2100 bioanalyzer for quality control. The double-stranded PCR products from previous step were heated denatured and circularized by the splint oligo sequence to get the final library. The single-strand circle DNA (ssCir DNA) was formatted as the final library. The final library was amplified with phi29 to make DNA nanoball (DNB) which had more than 300 copies of one molecular, DNBs were loaded into the patterned nanoarray, and pair end 150 bases reads were generated on BGIseq500 platform (BGI-Shenzhen, China). The sequences data reported in this study was archived in the Sequence Read Archive (SRA) with the accession number PRJNA879776 and PRJNA879113.

### RNA sequencing analysis

The sequencing data was filtered with SOAPnuke (v1.5.2) by removing reads containing sequencing adapter, removing reads whose low-quality base ratio (base quality less than or equal to 5) is more than 20%, removing reads whose unknown base (“N” base) ratio is more than 5%; afterwards, clean reads were obtained and stored in FASTQ format. The clean reads were mapped to the reference genome using HISAT2 (v2.0.4). Bowtie2 (v2.2.5) was applied to align the clean reads to the reference coding gene set, then expression level of gene was calculated by RSEM (v1.2.12). The heatmap was drawn by pheatmap (v1.0.8) according to the gene expression in different samples. Essentially, differential expression analysis was performed using the DESeq2 with |log2FC|> 1 (FC: fold change) and *p* ≤ 0.05.

### ChIP and re-ChIP

ChIP and re-ChIP were performed in LM3 cells as previously described [51, 52]. Briefly, cells were cross-linked using 1% formaldehyde, sonicated, precleared, and incubated with 5–10 μg of the appropriate antibody, followed by the addition of protein A/G magnetic beads. The beads were then washed in buffers with high- and low-salt concentrations, and DNA was eluted for PCR or qChIP assays. For re-ChIP, the beads were eluted with 20 mM dithiothreitol at 37 °C for 30 min, and the eluents were diluted 30-fold for further incubation with the appropriate secondary antibody and beads. The primers used are listed in Additional file 1: Table S2.

### Real-time quantitative RT–PCR

Total cellular RNA was extracted using an RNA-Quick Purification kit according to the manufacturer’s instructions (ES Science, Cat. RN001). Potential DNA contamination was mitigated using RNase-free DNase treatment. cDNA was prepared using a Transcriptor First-Strand cDNA Synthesis Kit (Roche, Cat. 0,489,703,001). Relative quantitative PCR was performed utilizing the ABI Prism 7500 sequence detection system (Applied Biosystems, Foster City, CA, USA) through the measurement of real-time SYBR Green fluorescence, and the results were obtained using the comparative Ct method (2^−∆∆Ct^) with GAPDH as an internal control. This experiment was performed in triplicate. The primers used are listed in Additional file 1: Table S3.

### EdU incorporation assay

LM3 or SK-HEP-1 cells transfected with SMYD3 siRNA or control siRNA were seeded into 6-well dishes at a density of 1 × 10^5^ cells/ml and allowed to adhere overnight. Next, the cells were cultured with 5-ethynyl-2’-deoxyuridine (EdU) for 2 h before detection. The proliferation rate of the cells was then evaluated using a Cell-Light EdU Cell Proliferation Detection kit (Beyotime, China, Cat. C0075) following the manufacturer’s instructions.

### TUNEL (TdT-mediated dUTP Nick-End Labeling) assay

LM3 cells transfected with SMYD3 siRNA or control siRNA were seeded into 6-well plates for 48 h. Then, cells were harvested and TUNEL assays were performed according to the manufacturer’s instructions (Promega, USA, Cat. TB235) using a fluorescence method. Positive and negative controls with the TUNEL assays were performed according to the instructions provided by the manufacturer.

### Cell invasion assay

Transwell chamber filters (Corning, NY, USA, Cat. 3422) were coated with Matrigel (BD, NY, USA, Cat. 356234). After infection with lentivirus, LM3 or SK-HEP-1 cells were suspended in serum-free DMEM or MEM media, and then, 2 × 10^4^ cells were seeded into the upper chamber in a volume of 300 μL. The chamber was cultured in a well containing 500 μL of DMEM or MEM media with 10% FBS at 37 °C for 18 h. Cells on the upper side of the membrane were removed using cotton swabs and those on the underside were stained and counted. Four high-powered fields were counted for each membrane.

### Colony formation assay

LM3 cells and SK-HEP-1 cells transfected with shSMYD3, shSCR, SMYD3, or vector were seeded into fresh 6-well plates at a density of 1000 cells/well and cultured in complete medium at 37 °C and 5% CO_2_. After 10–14 days, cells were fixed in methanol and stained with 0.1% crystal violet. The number of colonies was then manually counted.

### In vitro wound-healing assay

LM3 cells or SK-HEP1 cells in DMEM or MEM medium containing 10% FBS were seeded into 6-well plates. When the cells grew to about 100%, serum-free medium was replaced and cells were treated with 1 μg/ml mitomycin C for 1 h. The wounds were created using sterile pipette tips. Cells were washed with PBS and incubated in fresh medium without FBS. The cells were imaged after incubation at 37 °C for 24 or 36 h.

### Tumor xenografts

LM3 liver cancer cells stably transfected with shSMYD3 or shSCR were collected, and 4 × 10^6^ viable cells in 100 µl of PBS were subcutaneously injected into 6–8-week-old BALB/c nude mice (Vital River, Beijing, China). Eight animals per group were used in each experiment. Tumors were measured every 3 days using Vernier calipers, and tumor volume was calculated according to the following formula: π/6 × length × width^2^. All studies were approved by the Animal Care Committee of Zhejiang University.

### In vivo metastasis in NOD/SCID mice

LM3 cells that had been engineered to stably express firefly luciferase (PerkinElmer, Cat. 122799) was infected with lentiviruses carrying control shRNA or shSMYD3. A total 5 × 10^6^ cells of each type injected into the lateral tail vein of 6-week-old male NOD/SCID mice. For bioluminescence imaging, mice were abdominally injected with 200 mg/g D-luciferin in PBS. Ten minutes after injection, the mice were anesthetized, and a charge-coupled device camera (IVIS; Xenogen) was used to image the bioluminescence. All studies were approved by the Animal Care Committee of Zhejiang University.

### Statistical analysis

Results are shown as the mean ± SD unless otherwise indicated. Comparisons were performed using two-tailed paired *t*-tests based on a bidirectional hypothesis for continuous variables.

## Supplementary Information

**Additional file 1: Table S1-S3. Table S1.** siRNA and shRNA sequences. **Table S2. **The primers used in qChIP Assays. **Table S3.** Real-time quantitative primers used in this study.

**Additional file 2.** Raw date of gene expression and log2fc of differentially expressed genes (DEGs) between 5 pairs of HCC and adjacent normal tissues in RNA seq results.

**Additional file 3.** Mass spectrometry identification data for the interactomics of SMYD3 (the protein score equal to or higher than two).

**Additional file 4:** **Fig. S1. **PPI analysis of SMYD3-associated proteins.

**Additional file 5.** Raw date of gene expression and log2fc of differentially expressed genes whose expressions were altered upon SMYD3, MTA1 and MTA2 knockdown.

**Additional file 6: Fig. S2.** (a) LM3 cells were transfected with HDAC1 siRNA, HDAC2 siRNA, and the protein levels of H4ac were measured. H4 served as loading control for the western blot. (b) Western blot analysis was used to determine the protein expression in these cells using antibodies against the indicated proteins.

**Additional file 7: Fig. S3. **(a) Rescue experiment of EdU incorporation assays. LM3 cells were transfected with the indicated siRNA. EdU incorporation assays were performed using a fluorescence method. Representative images and statistical analyses are shown. (b) Rescue experiment of transwell invasion assays. LM3 cells were transfected with the indicated specific siRNA. The invaded cells were stained and quantified. The images represent one field under microscopy in each group. The efficiency of protein knockdown was verified by western blotting. *P < 0.05 and **P < 0.01 (two-tailed t-test). (c) The knockdown efficiencies of SMYD3 and IGFBP4 were confirmed by western blot.

**Additional file 8.** Images of the full immunoblots.

## Data Availability

Mass spectrometry data had been deposited in PRIDE [53], and data are available via ProteomeXchange with identifier PXD031971. RNA-seq data had been deposited in NCBI SRA (http://www.ncbi.nlm.nih.gov.sra) with the accession number PRJNA879776 [54] and PRJNA879113 [55]. All the other data generated or analyzed during this study are included in this published article and its supplementary information files.

## Abbreviations

HCC: Hepatocellular carcinoma
SMYD3: The SET and MYND domain-containing protein 3
EMT: Epithelial-mesenchymal transition
NuRD: Nucleosome remodeling and histone deacetylation
MTA: Metastasis-associated
IGFBP4: The insulin-like growth factor binding protein 4
HMT: Histone methyltransferase
FBS: Fetal bovine serum
IP: Immunoprecipitation
co-IP: Coimmunoprecipitation
ChIP: Chromatin immunoprecipitation
qRT-PCR: Quantitative reverse transcriptase polymerase chain reaction
GST: Glutathione S-transferase
EdU: 5-Ethynyl-2-deoxyuridine
TUNEL: TdT-mediated dUTP Nick-End Labeling
GEO: Gene Expression Omnibus
TCGA: The Cancer Genome Atlas
GSEAs: Gene set enrichment analyses
GO: Gene Ontology
BP: Biological process
CC: Cellular component
MF: Molecular function
