# Supplementary material for: SMYD3 associates with the NuRD (MTA1/2) complex to regulate transcription and promote proliferation and invasiveness in hepatocellular carcinoma cells
Source: BMC Biol. 2022 Dec 27;20:294. doi: 10.1186/s12915-022-01499-6 (PMC9795622; doi:10.1186/s12915-022-01499-6)
Supplement: Supplementary file 1 — Additional file 1: Table S1-S3. Table S1. siRNA and shRNA sequences. Table S2. The primers used in qChIP Assays. Table S3. Real-time quantitative primers used in this study. [file 12915_2022_1499_MOESM1_ESM.docx]

Supplementary Table S1. siRNA and shRNA sequences

| Control siRNA | TTCTCCGAACGTGTCACGT |
| --- | --- |
| SMYD3 siRNA-1 | GCAGAGTTGTCTTCAAACT |
| SMYD3 siRNA-1 | GAAGCCTTGTTCTATGGTA |
| IGFBP4 siRNA | GCAGAAGCACTTCGCCAAA |
| shSCR | TTCTCCGAACGTGTCACGT |
| shSMYD3 | GGATGGAGCACCTTCAGAATC |

Supplementary Table S2: The Primers used in qChIP Assays

| gene | strand | sequence |
| --- | --- | --- |
| IGFBP4 | F | CCCTCAAATCCTTCACTGCC |
| IGFBP4 | R | GCCCATTCAGACCCACTCG |
| CDH10 | F | AAAGTTCCCTGAAAGCA |
| CDH10 | R | CAATCTAATCCGCCTGT |
| CDH5 | F | GTGTTTCCTCGCCTCCCC |
| CDH5 | R | TGTGGCAACCCCATCCC |
| DLG5 | F | AACAGCCTATTTACCGCAGTTGA |
| DLG5 | R | CCCAGTCCAGAGGGTCCTAATC |
| CASP7 | F | CCGTGTTTGACTGACTCCC |
| CASP7 | R | AAGGCTGATCCGCTTCG |
| TRM4 | F | CACAGCCCTCTTCATTCCT |
| TRM4 | R | AGCCCTCGCCTTATTTG |
| NOLC1 | F | CACCCTCACGACGGATAA |
| NOLC1 | R | TCAGAGCAGCGGTTTCC |
| QGFR | F | GTTGGGGGATTCTCGGGG |
| QGFR | R | ATCCTCCTCGTCCTCCTCCC |
| DACH1 | F | CTCTGCTCCGAGTTCCTGGTTG |
| DACH1 | R | CGGCTGGACGAGTTGTTGTTGT |
| GRAMD4 | F | CCATCCTCACGTCCTCTTCAGC |
| GRAMD4 | R | GCCCTCCTCCAAGTCTCAGTCC |
| MOY9B | F | ATCCGCCATTTTCCCGCCTTCC |
| MOY9B | R | AGCCACCCCTCCTGCGTCTCCAC |
| LOXL4 | F | GACAAGGGCCAGGACTGCATCAT |
| LOXL4 | R | CAGGAACAGGCGAGTCACCCAGAG |
| PCDH9 | F | TCAGAGGGTCGCTCGCTTTCAG |
| PCDH9 | R | AGAGCCGGGTTCCATGAGGTGC |
| PRDM5 | F | GCAGGGTTCGTTCTCAGTAGTCG |
| PRDM5 | R | CCTTTGTCCAGCTCTGGGTTTT |
| GAPDH | F | GCCTCCAAACAGCCTTGC |
| GAPDH | R | GCCCTGACTTCCTCCACCT |

Supplementary Table S3. Real-time quantitative primers used in this study

| gene | strand | sequence |
| --- | --- | --- |
| SMYD3 | F | ACTGACTGAAGATAAGAAAGAGGG |
| SMYD3 | R | AGCAAAGAGATACTGGGATATAGG |
| IGFBP4 | F | CCTGCACACACTGATGCAC |
| IGFBP4 | R | CACCCTCGTCCTTGTCAGAG |
| CDH10 | F | CAGTAGAGCCAGAGTCAGAG |
| CDH10 | R | ACCACAGAAGTACCTACAACAG |
| CDH5 | F | CTCTCCACCATCATCAACTG |
| CDH5 | R | GCACTTCTTTCTTCATACGG |
| DLG5 | F | GCAAGCAGAAGAATGATGTC |
| DLG5 | R | AAGTCAATATCCTCCGTCTCC |
| CASP7 | F | GGTTCCAGGCTATTACTCGT |
| CASP7 | R | ATGGCTATTGACTGAAGTAGAG |
| TRM4 | F | AACTGAAAGAGGCTGAGACC |
| TRM4 | R | CTTACAAGAGAAGAGAAAGACCC |
| NOLC1 | F | AACCAGAAGCCAAAGATAACAC |
| NOLC1 | R | TTTAGGTACAGTCTCTGCCC |
| QGFR | F | CAGAAATGAGATCCGCTTCC |
| QGFR | R | TTTAAACACCTCGACCTCCC |
| MTA1 | F | AATATGGGAAGGATTTCACGGA |
| MTA1 | R | GATTTGGCTTGTTATAGTTGGG |
| MTA2 | F | GGGATGAGATGGAGGAATGG |
| MTA2 | R | ATATCACTCTGTTTCACGCC |
| DACH1 | F | GCTTCAACAGATAGTCTCAGG |
| DACH1 | R | CAGTCTTCCATCTTGTATTGTC |
| GRAMD4 | F | AGGTTCAGAGGTCACAAGAG |
| GRAMD4 | R | CCAATAAGGCAATTTCCAGGT |
| MOY9B | F | CTTATCCTTCCCTACAGCCT |
| MOY9B | R | CTGATATTCCTCCTGCTCCA |
| LOXL4 | F | CGTGGAGGTGAAGTATGAGG |
| LOXL4 | R | CCCAGACTTTCCTGTAGTAGTG |
| PCDH9 | F | ATCTCAAACCAGACACTCCA |
| PCDH9 | R | TGGCTTTCTTCATTCTCCTG |
| PRDM5 | F | GTCCCTATCAATGTCCTTACTG |
| PRDM5 | R | CCAAATCACAAACATCACACTG |
| GAPDH | F | TCCTCCTGTTTCATCCAAGC |
| GAPDH | R | TAGTAGCCGGGCCCTACTTT |
