# Supplementary figures and images for: SMYD3 associates with the NuRD (MTA1/2) complex to regulate transcription and promote proliferation and invasiveness in hepatocellular carcinoma cells
Source: BMC Biol. 2022 Dec 27;20:294. doi: 10.1186/s12915-022-01499-6 (PMC9795622; doi:10.1186/s12915-022-01499-6)

Fig. S2

a

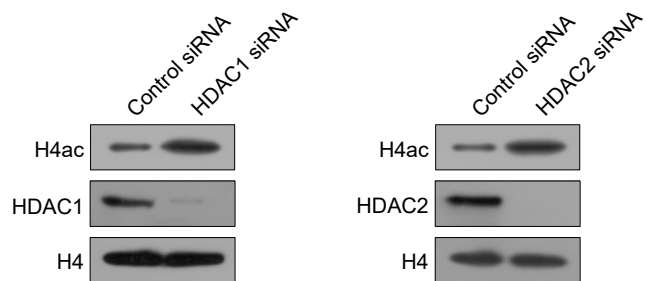

b

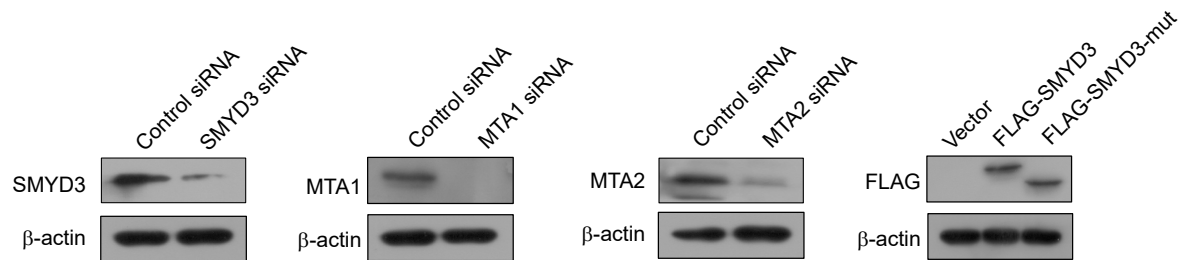

Supplement: Supplementary file 6 — Additional file 6: Fig. S2. (a) LM3 cells were transfected with HDAC1 siRNA, HDAC2 siRNA, and the protein levels of H4ac were measured. H4 served as loading control for the western blot. (b) Western blot analysis was used to determine the protein expression in these cells using antibodies against the indicated proteins. [file 12915_2022_1499_MOESM6_ESM.pdf]

Fig. S3

a

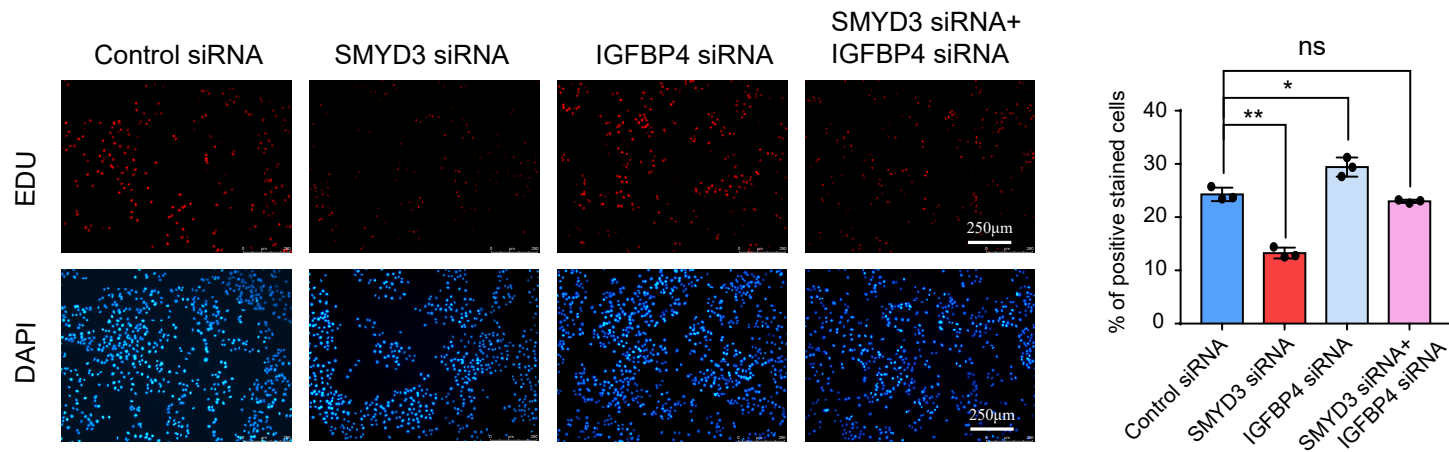

b

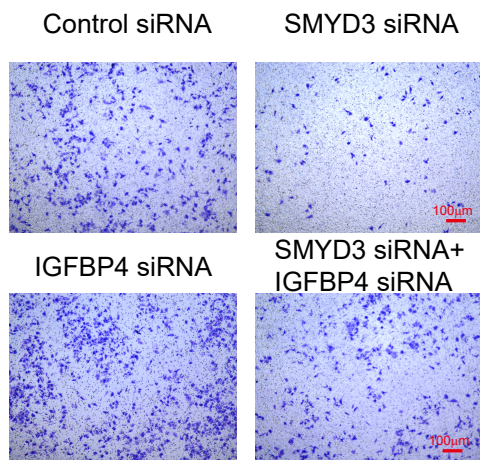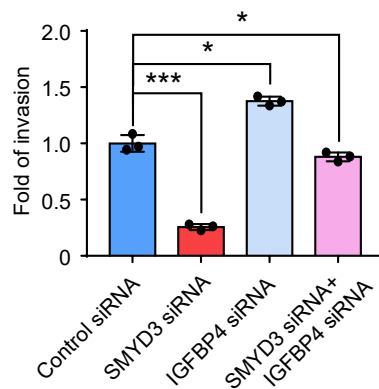

c

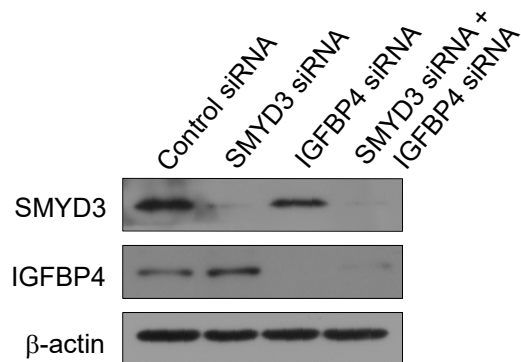

Supplement: Supplementary file 7 — Additional file 7: Fig. S3. (a) Rescue experiment of EdU incorporation assays. LM3 cells were transfected with the indicated siRNA. EdU incorporation assays were performed using a fluorescence method. Representative images and statistical analyses are shown. (b) Rescue experiment of transwell invasion assays. LM3 cells were transfected with the indicated specific siRNA. The invaded cells were stained and quantified. The images represent one field under microscopy in each group. The efficiency of protein knockdown was verified by western blotting. *P < 0.05 and **P < 0.01 (two-tailed t-test). (c) The knockdown efficiencies of SMYD3 and IGFBP4 were confirmed by western blot. [file 12915_2022_1499_MOESM7_ESM.pdf]
