# Supplementary material for: SMYD3 associates with the NuRD (MTA1/2) complex to regulate transcription and promote proliferation and invasiveness in hepatocellular carcinoma cells
Source: BMC Biol. 2022 Dec 27;20:294. doi: 10.1186/s12915-022-01499-6 (PMC9795622; doi:10.1186/s12915-022-01499-6)

Figure 1 raw data

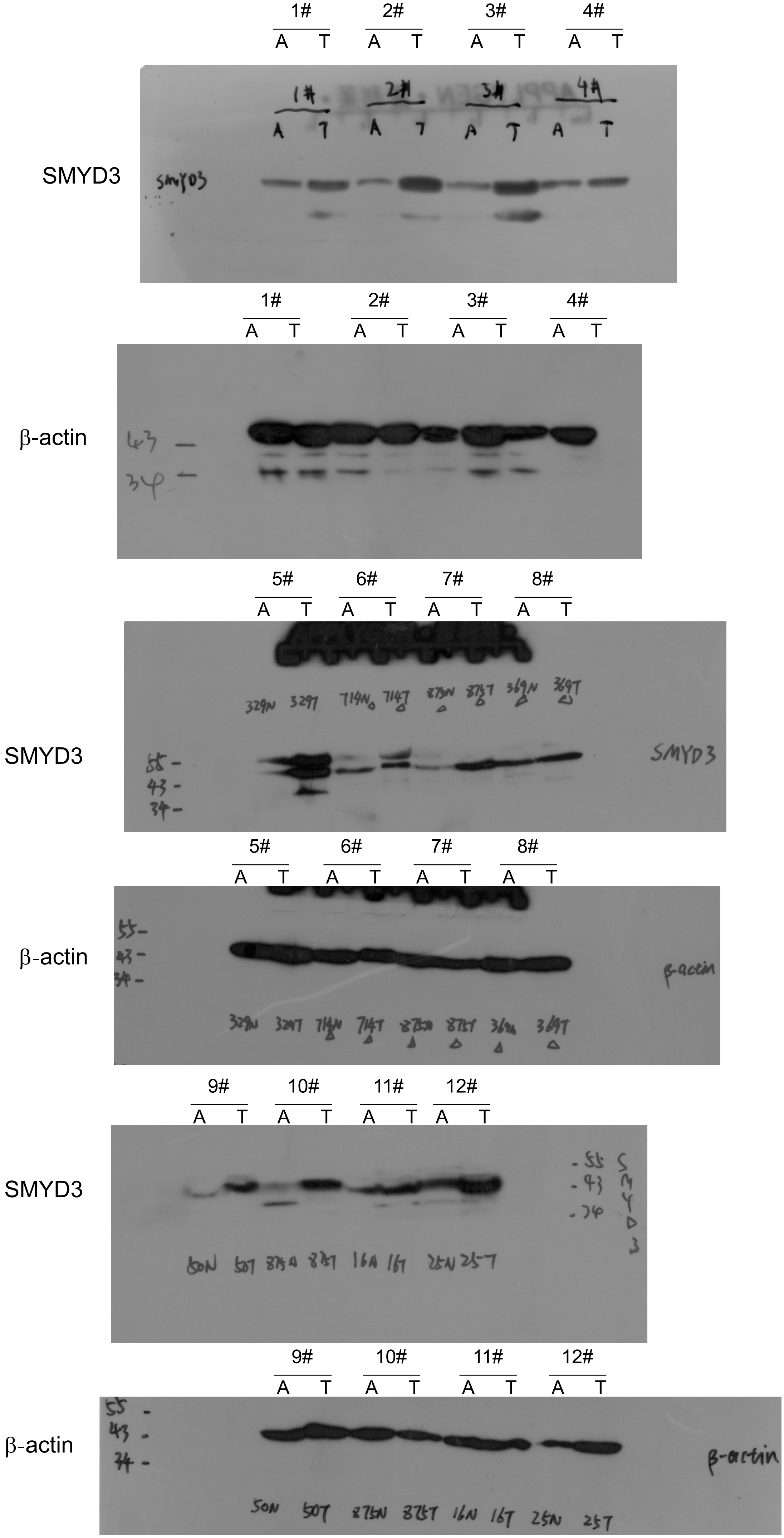

Figure 2 and 3 raw data

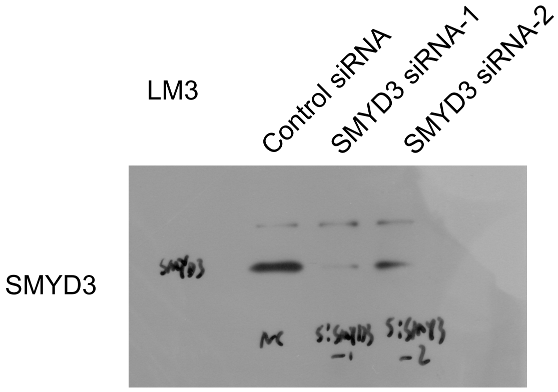

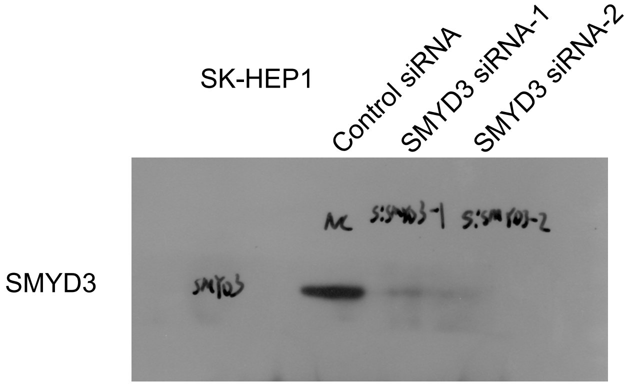

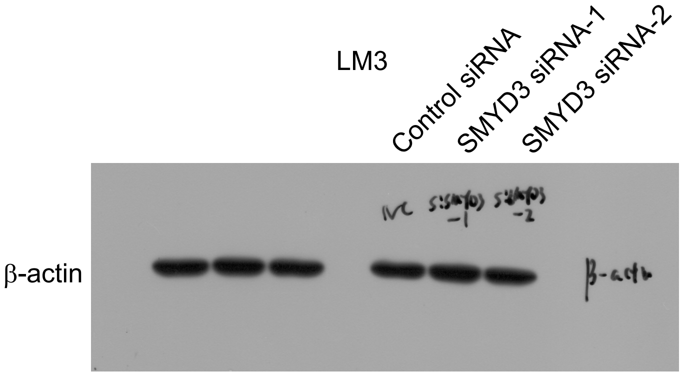

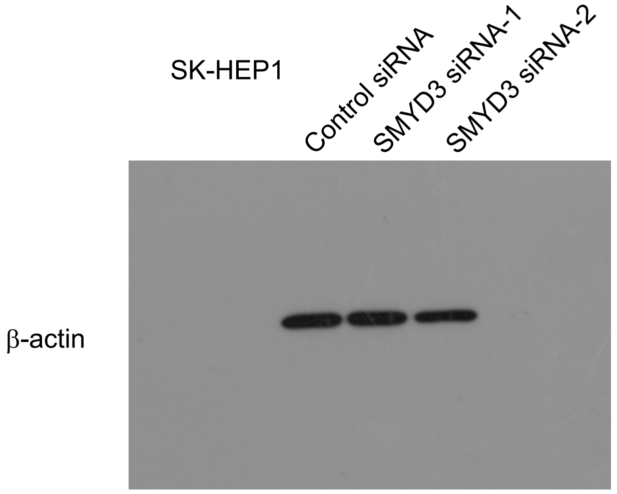

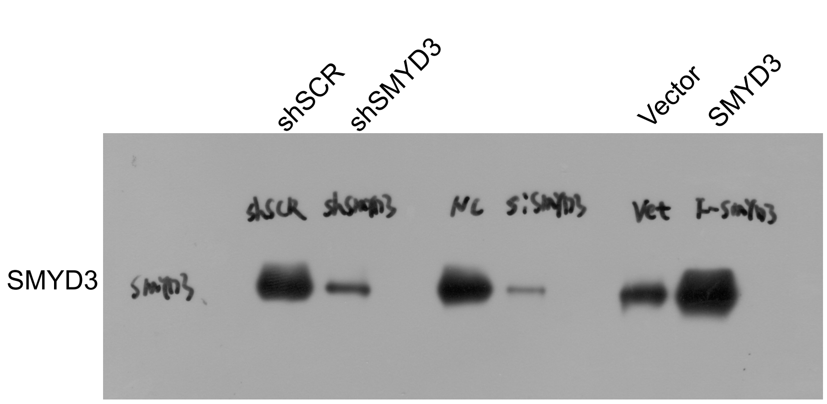

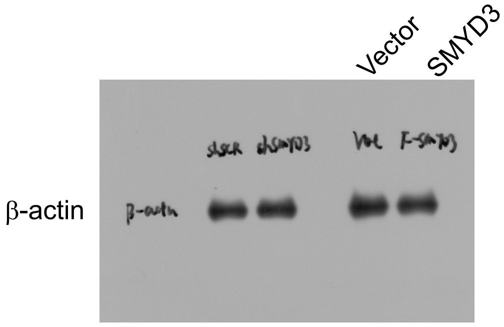

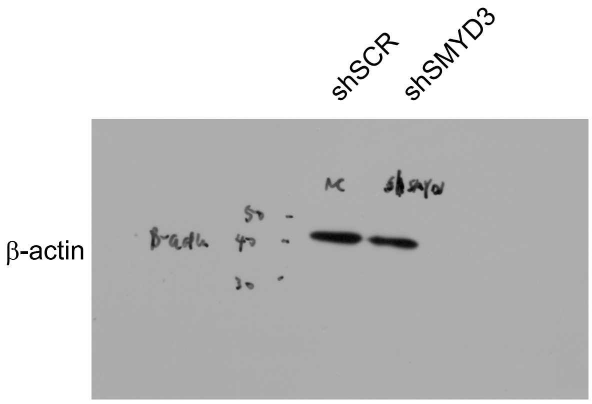

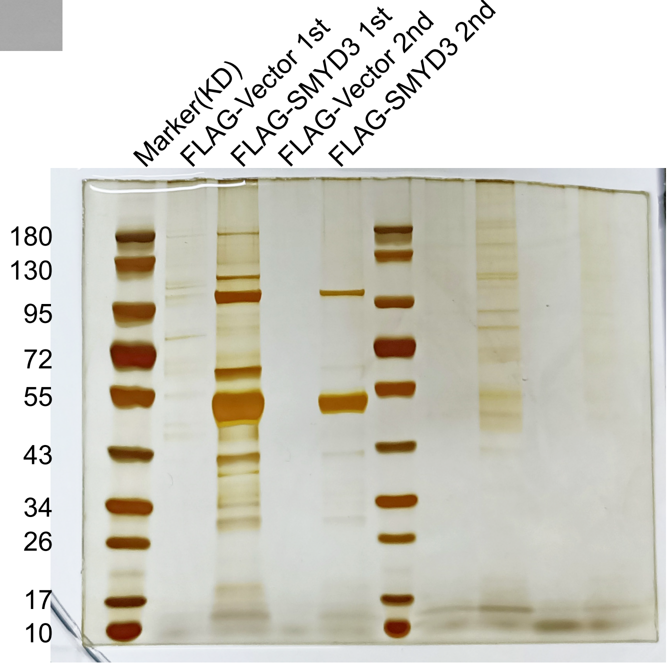

Figure 4A and B raw data

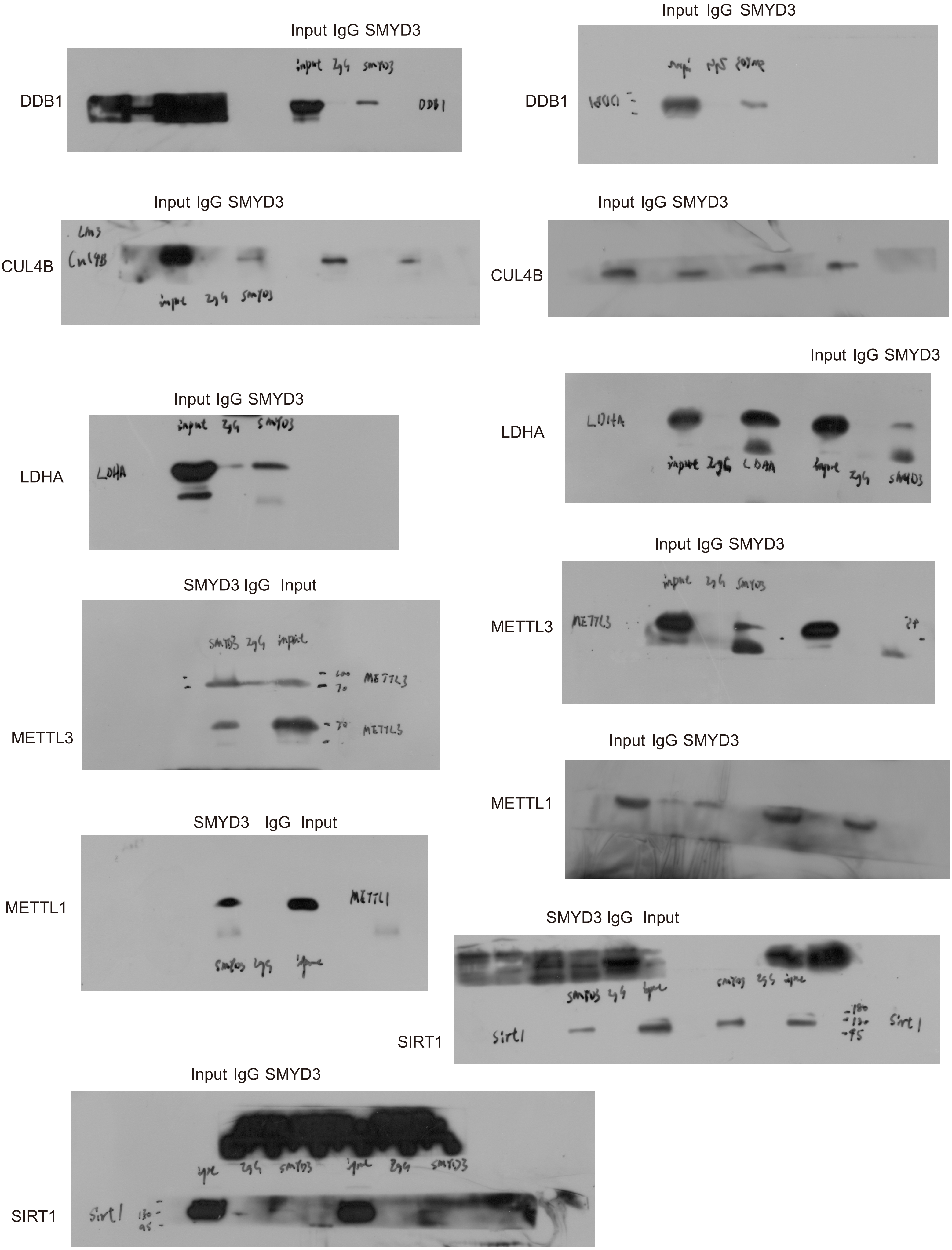

Figure 4C and D raw data

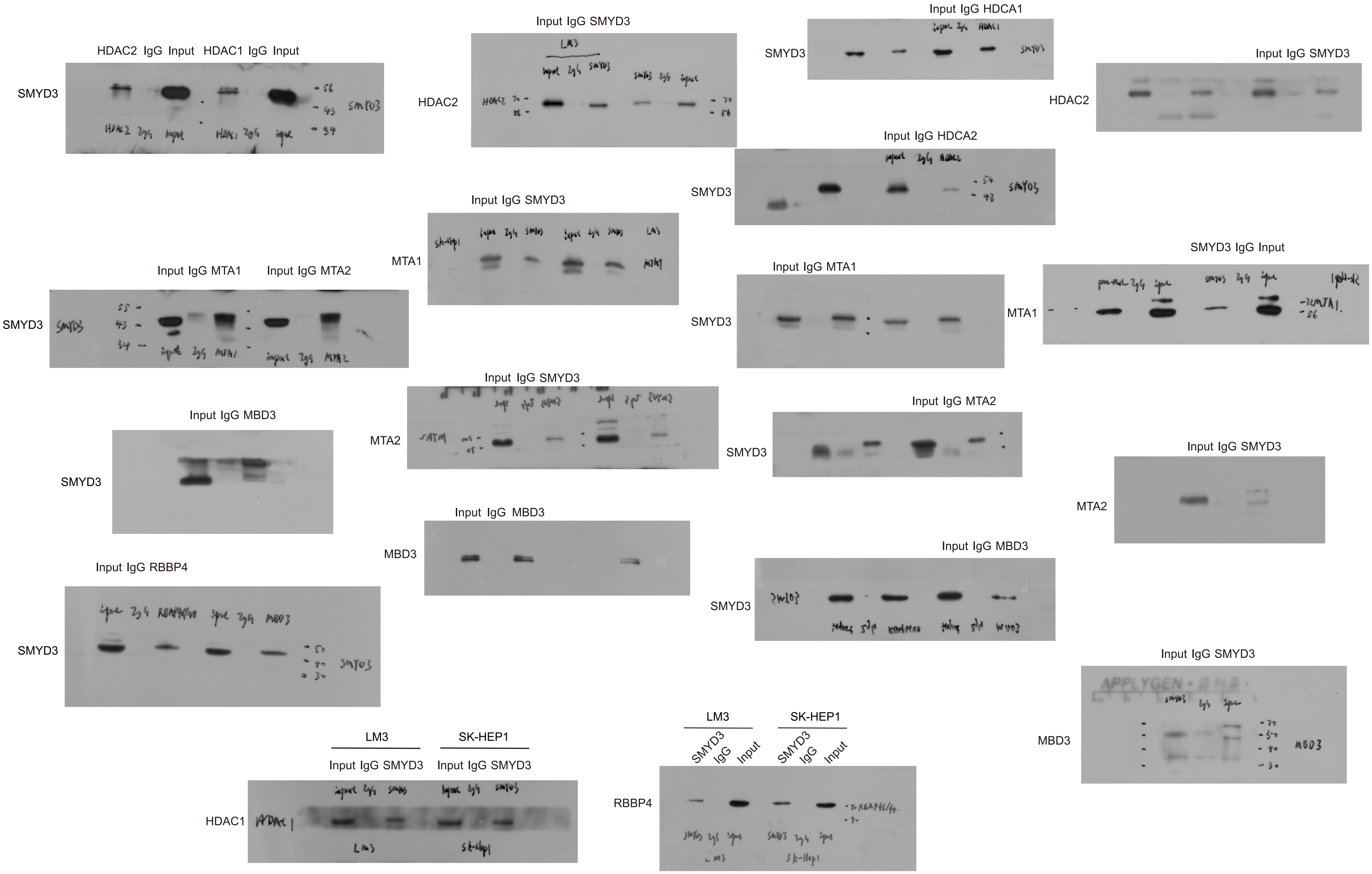

Figure 6 and 7 raw data

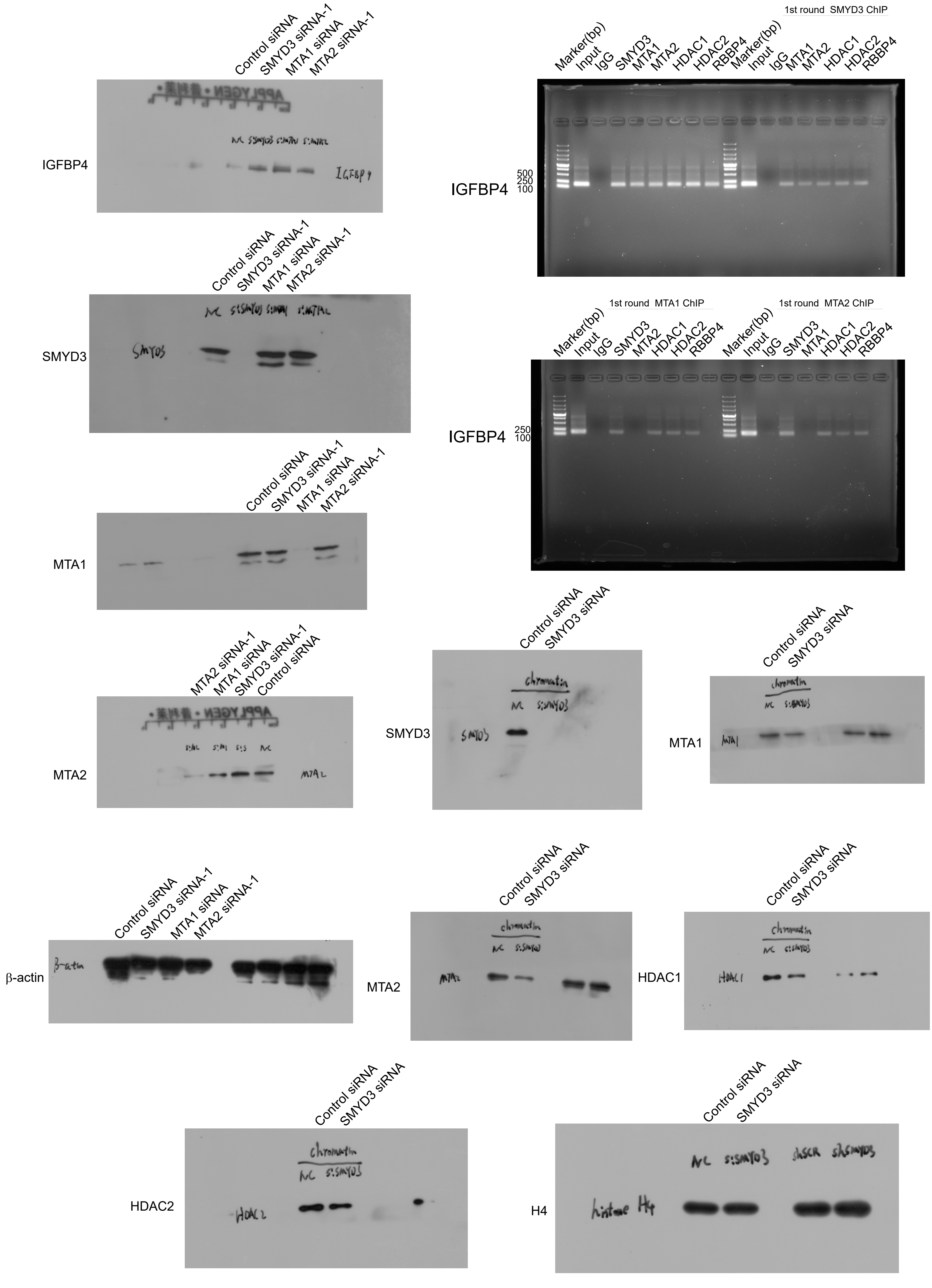

Figure S2 and S3 raw data

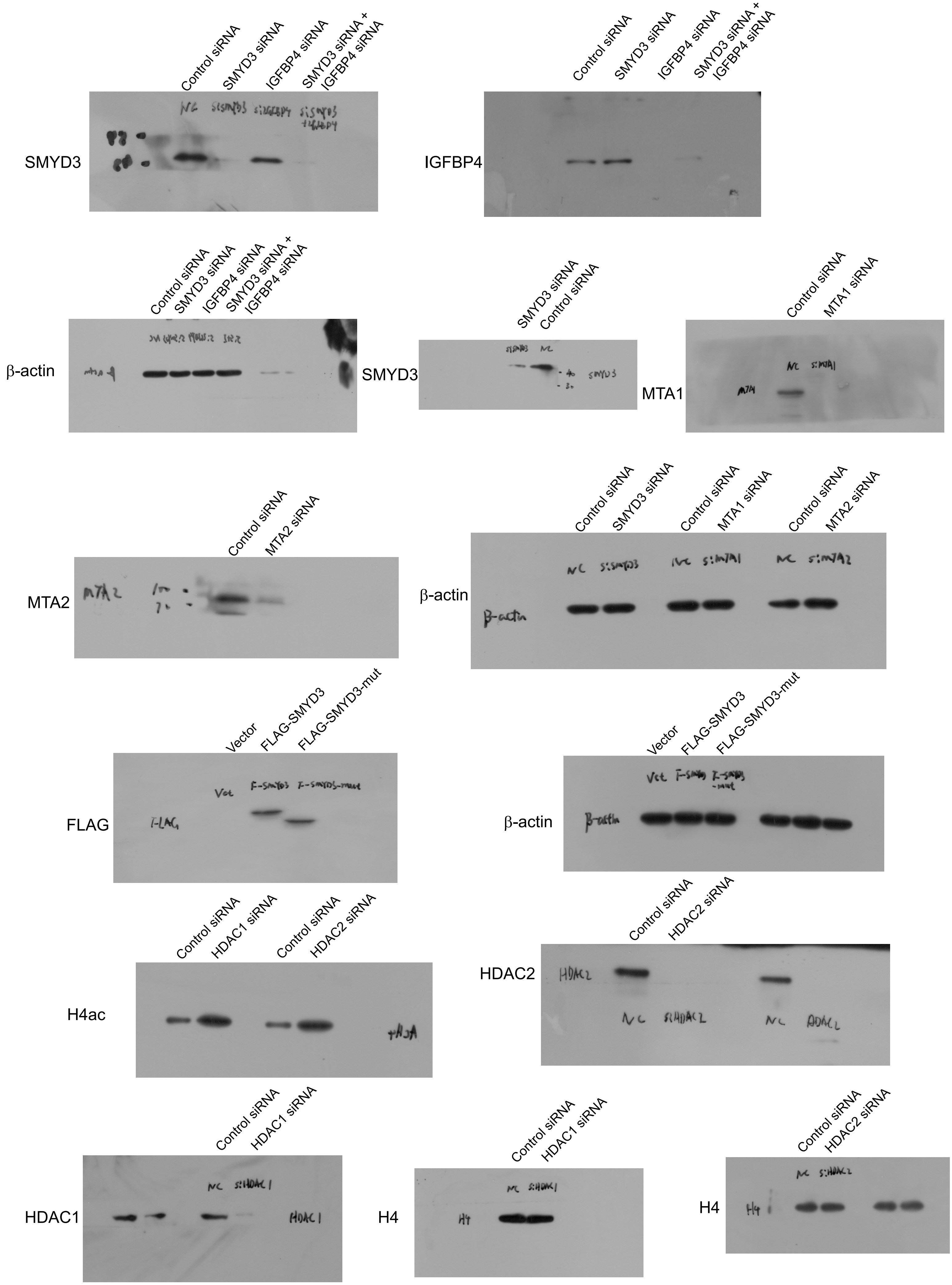

Supplement: Supplementary file 8 — Additional file 8. Images of the full immunoblots. [file 12915_2022_1499_MOESM8_ESM.pdf]
