# Supplementary material for: SMYD3 associates with the NuRD (MTA1/2) complex to regulate transcription and promote proliferation and invasiveness in hepatocellular carcinoma cells
Source: BMC Biol. 2022 Dec 27;20:294. doi: 10.1186/s12915-022-01499-6 (PMC9795622; doi:10.1186/s12915-022-01499-6)
Supplement: Supplementary file 4 — Additional file 4: Fig. S1. PPI analysis of SMYD3-associated proteins. [file 12915_2022_1499_MOESM4_ESM.pdf]

Fig. S1

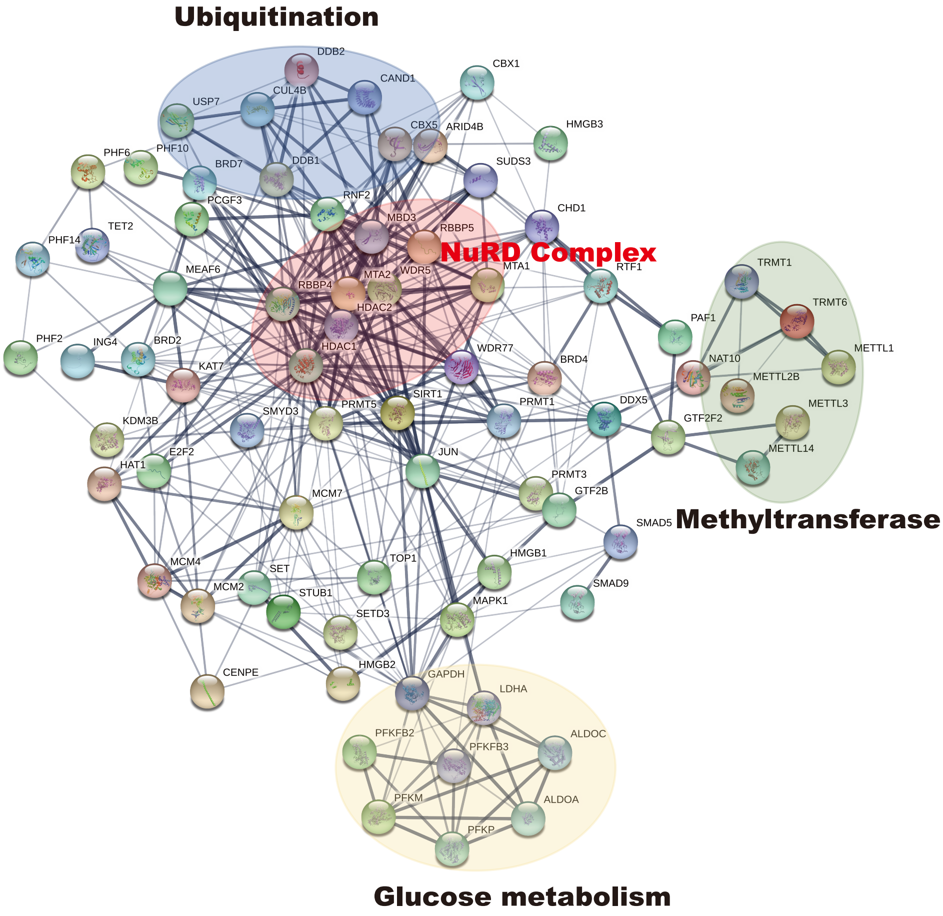
Ubiquitination
NuRD Complex
Methyltransferase
Glucose metabolism
DDB2
CAND1
CUL4B
USP7
CBX1
CBX5
ARID4B
HMGB3
PHF6
PHF10
BRD7
DDB1
SUDS3
RNF2
PCGF3
CHD1
MBD3
RBBP5
TET2
PHF14
MEAF6
MTA1
RTF1
TRMT1
MTA2
WDR5
RBBP4
HDAC2
TRMT6
PAF1
METTL1
PHF2
ING4
BRD2
HDAC1
WDR77
NAT10
BRD4
METTL2B
KAT7
SIRT1
DDX5
METTL3
SMYD3
PRMT5
PRMT1
GTF2F2
KDM3B
E2F2
JUN
METTL14
HAT1
PRMT3
MCM7
GTF2B
SMAD5
HMGB1
TOP1
MCM4
SET
SMAD9
MCM2
STUB1
MAPK1
SETD3
CENPE
HMGB2
GAPDH
LDHA
PFKFB2
ALDOC
PFKFB3
PFKM
ALDOA
PFKP
